# Multimodal computer vision and machine learning for predictive monitoring of planar-flow casting of amorphous steel foils

**DOI:** 10.1371/journal.pone.0347230

**Published:** 2026-11-03

**Authors:** Ritaban Dutta, Michael Kellam, Daniel Liang

**Affiliations:** 1 CSIRO Technology, Hobart, Tasmania, Australia; 2 CSIRO Technology, Clayton South, Victoria, Australia; University of Vigo, SPAIN

## Abstract

**Background:**

Planar-flow casting (PFC) is widely used for manufacturing amorphous steel foils, where small variations in process conditions can rapidly affect foil quality. Existing monitoring approaches primarily rely on heuristic measurements or offline inspection, providing limited capability for quantitative process prediction and early identification of unstable operating conditions.

**Methodology/Principal Findings:**

We present a multimodal computer vision and machine learning framework for predictive monitoring of industrial PFC using synchronised visible and infrared imagery acquired during full-scale production. More than 300,000 RGB and thermal image frames collected from approximately 200 production runs were processed to extract process-informed geometric, thermal and texture descriptors, including ridge density, ridge width, reflection characteristics and thermal-gradient features. Conventional image-processing baselines were compared with gradient-boosted decision trees (XGBoost) and a multimodal deep-learning fusion model combining RGB, infrared and process metadata. Both learning-based approaches substantially improved prediction of the nozzle-to-wheel gap compared with conventional computer-vision heuristics, achieving mean absolute errors of approximately 0.01 mm and R^2^ values of approximately 0.97. In contrast, prediction of the optical-flow-based melt-flow proxy remained substantially more challenging, indicating that this variable is influenced by factors not fully represented in the available imaging modalities. Feature-importance, calibration and ablation analyses demonstrated that ridge-density and thermal-gradient descriptors contributed most strongly to predictive performance, while complementary analyses showed that unstable production exhibited broader ridge-width distributions and larger edge-to-centre thermal gradients than stable operation.

**Conclusions/Significance:**

The proposed framework provides accurate, interpretable and non-contact predictive monitoring for industrial planar-flow casting while offering quantitative insights into process behaviour beyond conventional image-based inspection. The results demonstrate the value of combining multimodal imaging with machine learning for intelligent monitoring of advanced manufacturing processes and establish a foundation for future real-time process control and digital manufacturing applications.

## 1. Introduction

Planar-flow casting (PFC) is the principal manufacturing process for producing amorphous steel foils used in high-efficiency electrical machines, transformers, power electronics, and other advanced electromagnetic applications. During PFC, molten alloy is ejected through a narrow nozzle onto a rapidly rotating copper wheel, where cooling rates exceeding 10^5^–10^6^ K s^-1^ suppress crystallisation and enable the formation of an amorphous microstructure. Achieving stable ribbon formation requires precise control of multiple interacting process variables, including the nozzle–wheel gap, melt delivery conditions, wheel speed, and thermal behaviour at the wheel–melt interface. Even relatively small deviations in these parameters can lead to variations in ribbon geometry, surface morphology, and overall product quality [1–5].

Despite decades of industrial development, reliable monitoring of PFC remains challenging because the critical physical phenomena occur within milliseconds within a confined, high-temperature environment. Conventional monitoring approaches typically rely on discrete process measurements, operator observations, or post-process inspection of the produced ribbon. Although these approaches provide valuable operational information, they offer limited capability for continuous quantitative assessment of transient melt-delivery behaviour or for identifying process instabilities before they affect product quality.

Recent advances in computer vision and machine learning have created new opportunities for intelligent monitoring of advanced manufacturing processes. Image-based monitoring has been successfully applied in additive manufacturing, welding, rolling, and continuous casting to extract quantitative information from high-speed visual and thermal data for process assessment, defect detection, and quality prediction. Similar opportunities exist for planar-flow casting because the process naturally generates observable visual and thermal signatures, including melt-pool reflections, ribbon morphology, surface ridges, and temperature distributions, that evolve in response to changing operating conditions. However, relatively few studies have investigated the integration of multimodal imaging with machine learning for quantitative monitoring of PFC, and existing approaches have generally relied on single sensing modalities, relatively small experimental datasets, or qualitative interpretation [6–12].

An additional challenge is balancing predictive performance with physical interpretability. Deep-learning models can achieve excellent predictive accuracy but often provide limited insight into the physical mechanisms underlying their predictions. In contrast, feature-based machine-learning approaches built upon physics-informed image descriptors offer the potential to achieve competitive predictive performance while retaining interpretability. For industrial manufacturing, understanding *why* a prediction is made is often as important as the prediction itself, particularly when process engineers need to identify the physical factors associated with stable or unstable casting behaviour [13–20].

In this study, we present a multimodal computer vision and machine learning framework for predictive monitoring of planar-flow casting using synchronised front-view RGB, side-view RGB, infrared imaging, and process metadata collected from approximately 200 casting experiments (Table 1). The overall experimental workflow is illustrated in Fig 1, where multimodal image acquisition, automated computer-vision processing, feature extraction, and machine-learning modelling are integrated into a unified analytical framework. Following image quality control and region-of-interest extraction, 27 physics-informed descriptors representing reflection behaviour, ridge morphology, thermal characteristics, and process metadata were extracted from approximately 300,000 synchronised image frames and organised into a structured dataset for subsequent analysis (Table 2). The image frames were aggregated into temporally aligned feature vectors, resulting in approximately 8,700 samples for machine-learning analysis.

**Table 1:** Summary of the experimental dataset, imaging modalities, prediction targets, and modelling framework used in this study. The dataset comprised approximately 200 casting runs and 300,000 synchronised RGB and infrared image frames. Physics-informed descriptors extracted from the multimodal image data were used to develop and evaluate baseline heuristic models, feature-based XGBoost models, and multimodal deep-learning fusion models under group-wise cross-validation by casting run.

Category	Description
**Manufacturing process**	Planar-flow casting (PFC) of amorphous steel foil
**Experimental platform**	Laboratory-scale PFC system with synchronised RGB and infrared (IR) imaging
**Casting runs**	Approximately 200 production runs
**Total image frames**	Approximately 300,000 synchronised RGB and IR frames
**Total dataset size**	Approximately 20 GB
**Imaging modalities**	Front-view RGB, side-view RGB, and infrared (IR) imaging
**Image resolution**	RGB: 2048 × 2048 pixels; IR: 320 × 256 pixels
**Frame rate**	25 frames per second (fps)
**Feature dataset**	Approximately 8,700 aggregated samples
**Physics-informed descriptors**	27 image-derived descriptors
**Primary prediction target**	Nozzle–wheel gap (mm)
**Secondary prediction target**	Flow proxy (% change in optical flow velocity)
**Recorded process metadata**	Wheel speed, melt temperature, nozzle geometry, nozzle–wheel gap, and flow proxy
**Predictive models evaluated**	Baseline heuristic models, XGBoost, and multimodal deep-learning fusion
**Model validation**	Group-wise cross-validation by casting run

**Table 2:** Physics-informed descriptors extracted from synchronised RGB and infrared image sequences. Ridge descriptors quantify surface morphology, reflection descriptors characterise the nozzle–wheel interaction region, thermal descriptors represent heat-transfer behaviour, and process variables provide contextual operating information. These descriptors formed the input feature set for the feature-based XGBoost models and were also incorporated into the multimodal deep-learning framework.

Feature category	Descriptor	Unit	Physical interpretation	Primary application
**Ridge**	Ridge density	1/mm	Number of surface ridges per unit foil width; indicator of melt-flow instability	Gap prediction, **Process stability assessment**
	Mean ridge width	mm	Average width of surface ridges reflecting ribbon uniformity	Gap prediction, **Process stability assessment**
	Ridge-width standard deviation	mm	Variability in ridge width indicating localised process fluctuations	Gap prediction
	Ridge spacing	mm	Mean spacing between adjacent ridges; measure of solidification uniformity	**Process stability assessment**
	Ridge clustering index	–	Degree of local ridge aggregation associated with unstable casting	**Process stability assessment**
**Reflection**	Reflection intensity	Normalised intensity	Optical reflection from the melt–wheel interface used as an indirect indicator of nozzle–wheel gap	Gap prediction
	Reflection height	mm (calibrated)	Apparent height of the reflection profile after geometric calibration	Gap prediction
	Reflection profile variation	Normalised intensity	Spatial variability of the reflection signal along the ribbon width	Gap prediction
**Thermal**	Edge-to-centre temperature gradient	°C	Temperature difference between foil edge and centre representing heat-transfer imbalance	**Process stability assessment**
	Thermal-gradient variance	°C^2^	Spatial variability of thermal gradients across the foil width	Gap prediction, **Process stability assessment**
	Thermal asymmetry	°C	Left–right temperature imbalance across the ribbon	**Process stability assessment**
	Hot-area fraction	%	Percentage of foil area above a selected temperature threshold	Flow-proxy estimation
**Process**	Wheel speed	rpm	Cooling-wheel rotational speed recorded during casting	Context variable
	Melt temperature	°C	Nominal melt temperature during casting	Context variable
	Nozzle geometry	Categorical	Slit configuration defining melt-delivery characteristics	Context variable
	Previous gap estimate	mm	Previous-frame gap value incorporated for temporal continuity	Gap prediction
	Previous flow proxy	% change in optical flow velocity	Previous-frame flow estimate used for temporal prediction	Flow-proxy prediction

**Fig 1:**
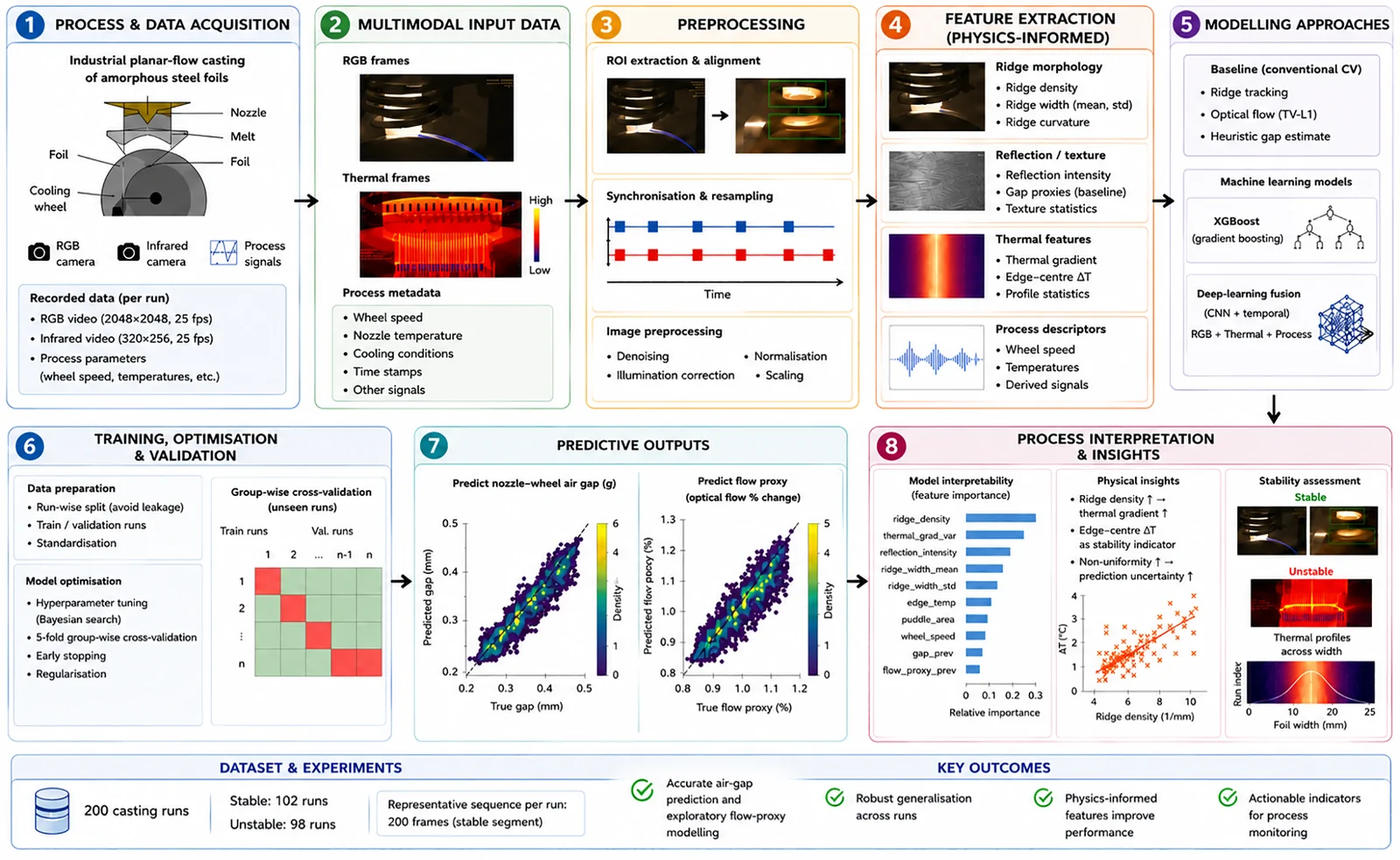
Overview of the proposed multimodal computer-vision and machine-learning framework for predictive monitoring of planar-flow casting. The workflow integrates eight principal stages: (1) planar-flow casting and multimodal data acquisition; (2) synchronised RGB, infrared and process-data inputs; (3) image preprocessing, region-of-interest extraction and temporal alignment; (4) extraction of physics-informed ridge morphology, reflection, thermal and process descriptors; (5) predictive modelling using analytical computer-vision baselines, feature-based XGBoost and multimodal deep-learning fusion; (6) model training, optimisation and group-wise cross-validation by casting run; (7) prediction of nozzle–wheel gap and the optical-flow-based flow proxy; and (8) model interpretation and assessment of the physical characteristics associated with casting stability. The framework links multimodal sensing with interpretable feature extraction, predictive modelling and process-level interpretation while maintaining separation of casting runs during model validation.

Three complementary modelling strategies were evaluated. Analytical computer-vision baselines were first established to quantify the predictive capability of conventional heuristic approaches (Fig 2). These results were compared with feature-based XGBoost models (Fig 3) and a multimodal deep-learning fusion framework (Fig 4) using group-wise cross-validation to ensure complete separation of casting runs between training and validation datasets. The physical significance of the extracted ridge and thermal descriptors was subsequently investigated through quantitative morphology and thermal analyses (Fig 5), while feature importance and systematic ablation experiments were used to evaluate the contribution of individual descriptor groups to predictive performance (Fig 6). Finally, the proposed framework was qualitatively demonstrated using factory-acquired casting sequences to examine the consistency of the observed visual and thermal characteristics under industrial operating conditions (Fig S1 in S1 File).

**Fig 2:**
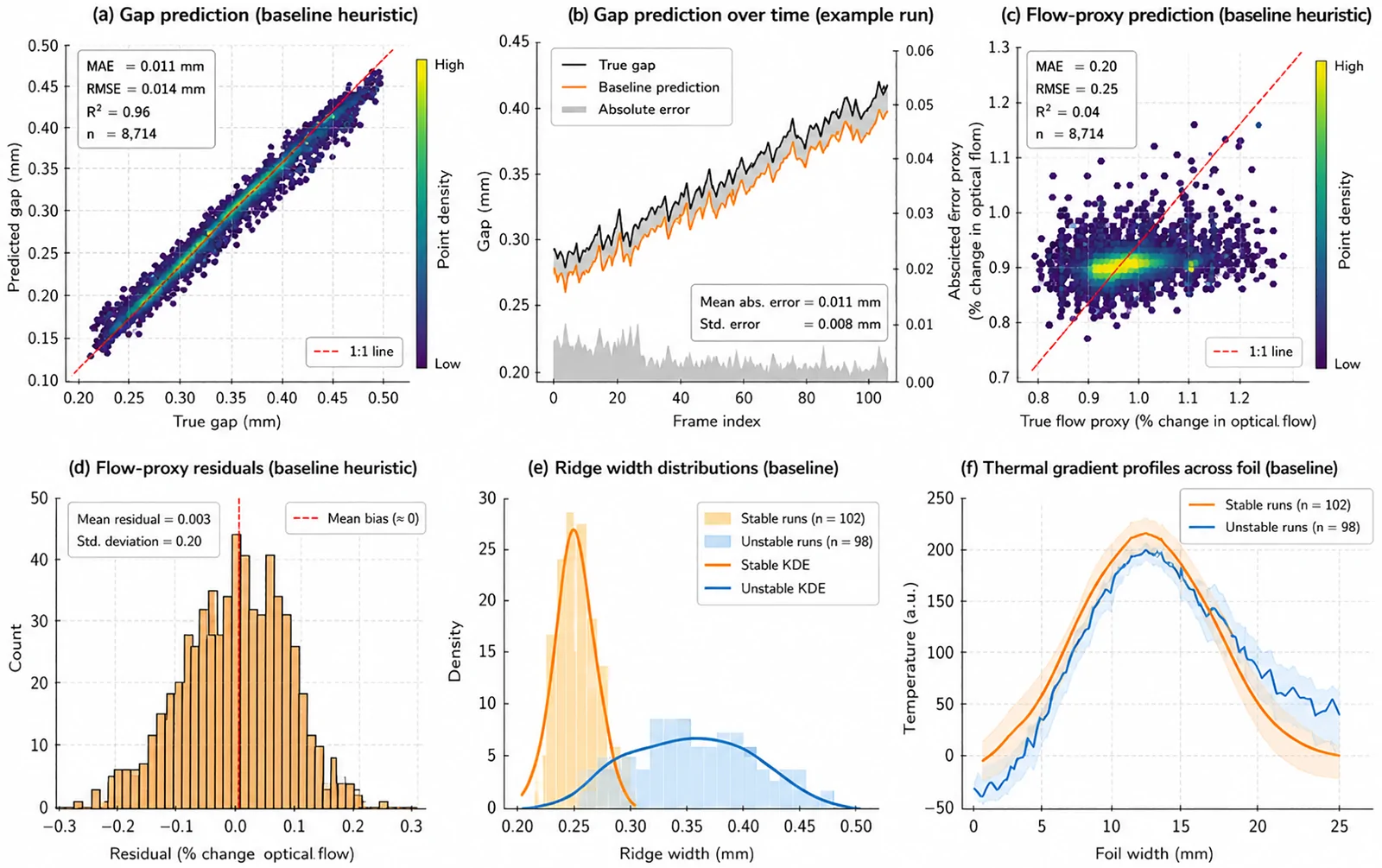
Performance of the analytical computer-vision baseline models for prediction of nozzle–wheel gap and optical-flow-based flow proxy. (a) Relationship between measured and predicted nozzle–wheel gap values obtained using the analytical computer-vision baseline model. (b) Representative time-series comparison of measured and predicted nozzle–wheel gap during an individual casting run. (c) Measured versus predicted optical-flow-based flow-proxy values, demonstrating limited predictive capability using conventional analytical image processing. (d) Distribution of prediction residuals for nozzle–wheel gap estimation. Performance metrics (MAE, RMSE and R^2^) are reported within the corresponding panels. The analytical baseline provided a useful reference for subsequent machine-learning models by capturing the dominant geometric characteristics of the casting process while demonstrating limited capability for modelling the more complex melt-flow behaviour represented by the optical-flow-based flow proxy.

**Fig 3:**
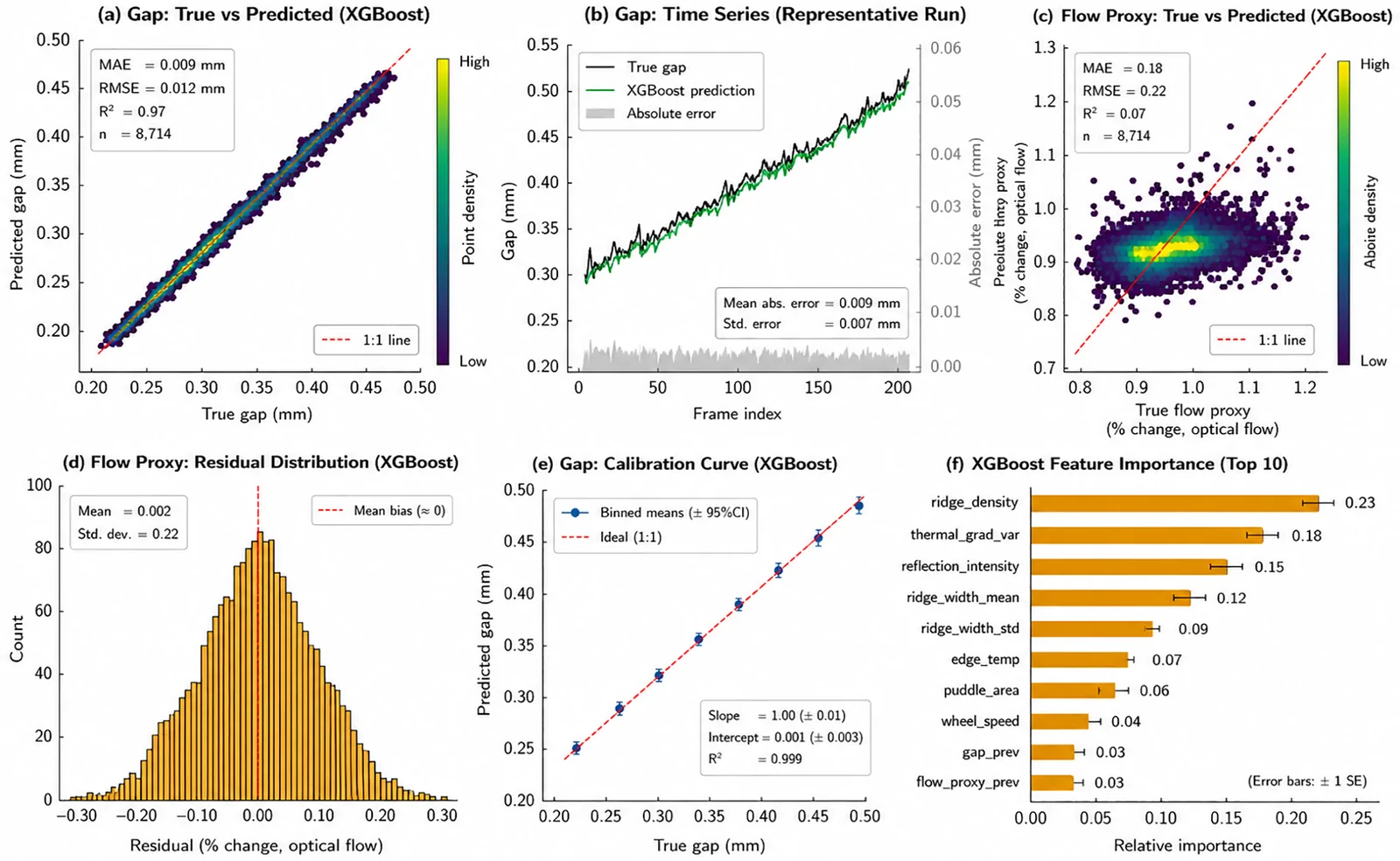
Performance and interpretability of the feature-based XGBoost model for prediction of nozzle–wheel gap and optical-flow-based flow proxy. (a) Relationship between measured and predicted nozzle–wheel gap values obtained using the feature-based XGBoost model. (b) Representative time-series comparison of measured and predicted nozzle–wheel gap during an individual casting run. (c) Relative importance of the physics-informed descriptors, highlighting the contributions of ridge morphology, thermal characteristics, reflection behaviour and process variables to model prediction. (d) Calibration plot comparing predicted and measured nozzle–wheel gap values across the full operating range. (e) Distribution of prediction residuals demonstrating the stability and absence of significant systematic bias in the XGBoost predictions. (f) Measured versus predicted optical-flow-based flow-proxy values. Quantitative performance metrics (MAE, RMSE and R^2^) are reported within the corresponding panels. The results demonstrate that physics-informed feature engineering substantially improves prediction of the nozzle–wheel gap while maintaining model interpretability through explicit feature-importance analysis, although prediction of the optical-flow-based flow proxy remains comparatively limited.

**Fig 4:**
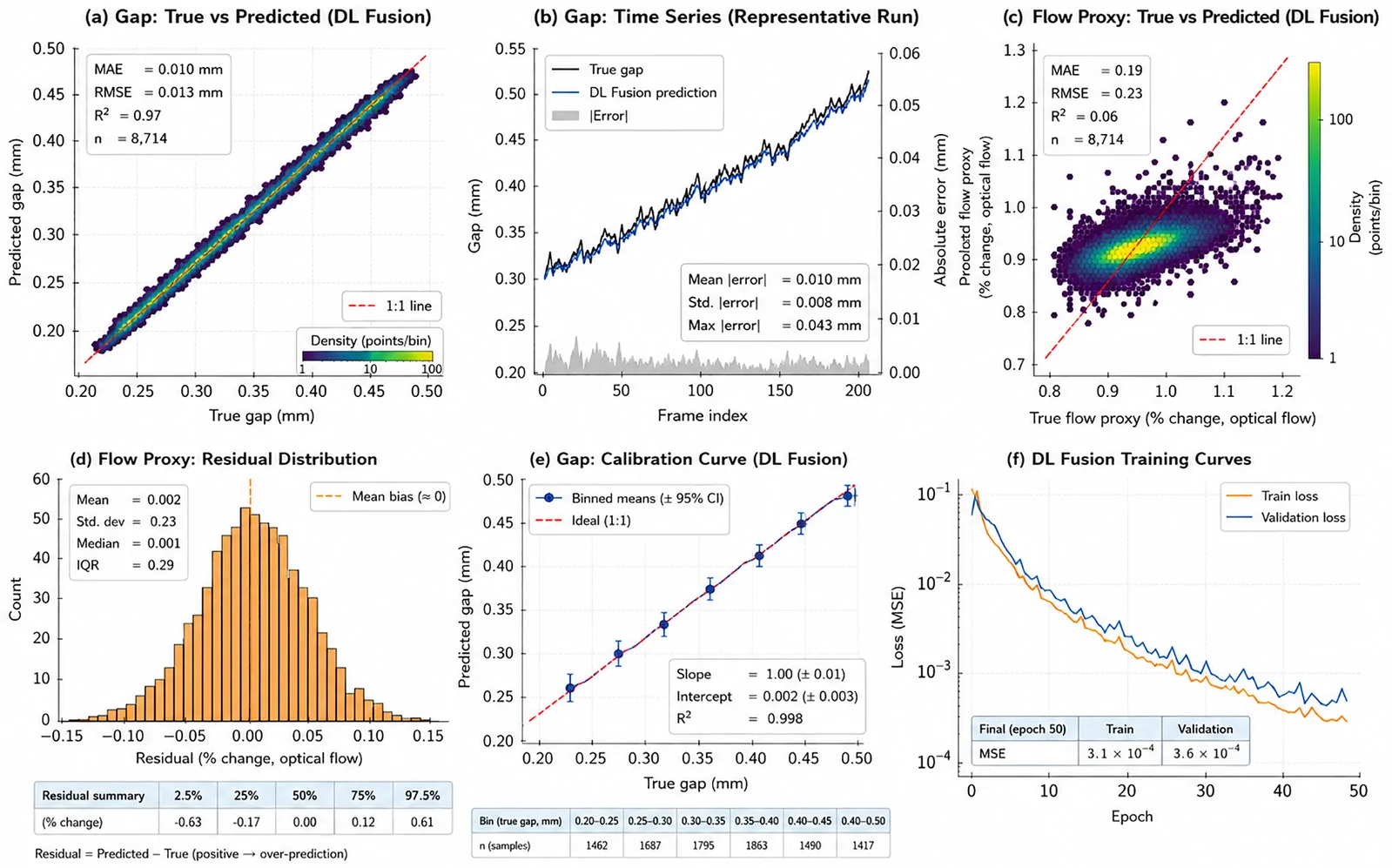
Performance of the multimodal deep-learning fusion model for prediction of nozzle–wheel gap and optical-flow-based flow proxy. (a) Relationship between measured and predicted nozzle–wheel gap values obtained using the multimodal deep-learning fusion model. (b) Representative time-series comparison of measured and predicted nozzle–wheel gap during an individual casting run. (c) Training and validation loss curves demonstrating stable model convergence during network optimisation. (d) Calibration plot comparing predicted and measured nozzle–wheel gap values across the full operating range. (e) Distribution of prediction residuals showing consistent prediction accuracy with minimal systematic bias. (f) Measured versus predicted optical-flow-based flow-proxy values. Quantitative performance metrics (MAE, RMSE and R^2^) are reported within the corresponding panels. The multimodal deep-learning fusion model achieved prediction accuracy comparable to the feature-based XGBoost model while directly learning complementary spatial, thermal and process representations from synchronised multimodal data. Consistent calibration, stable convergence and narrow residual distributions demonstrate robust model generalisation across the evaluated casting conditions.

**Fig 5:**
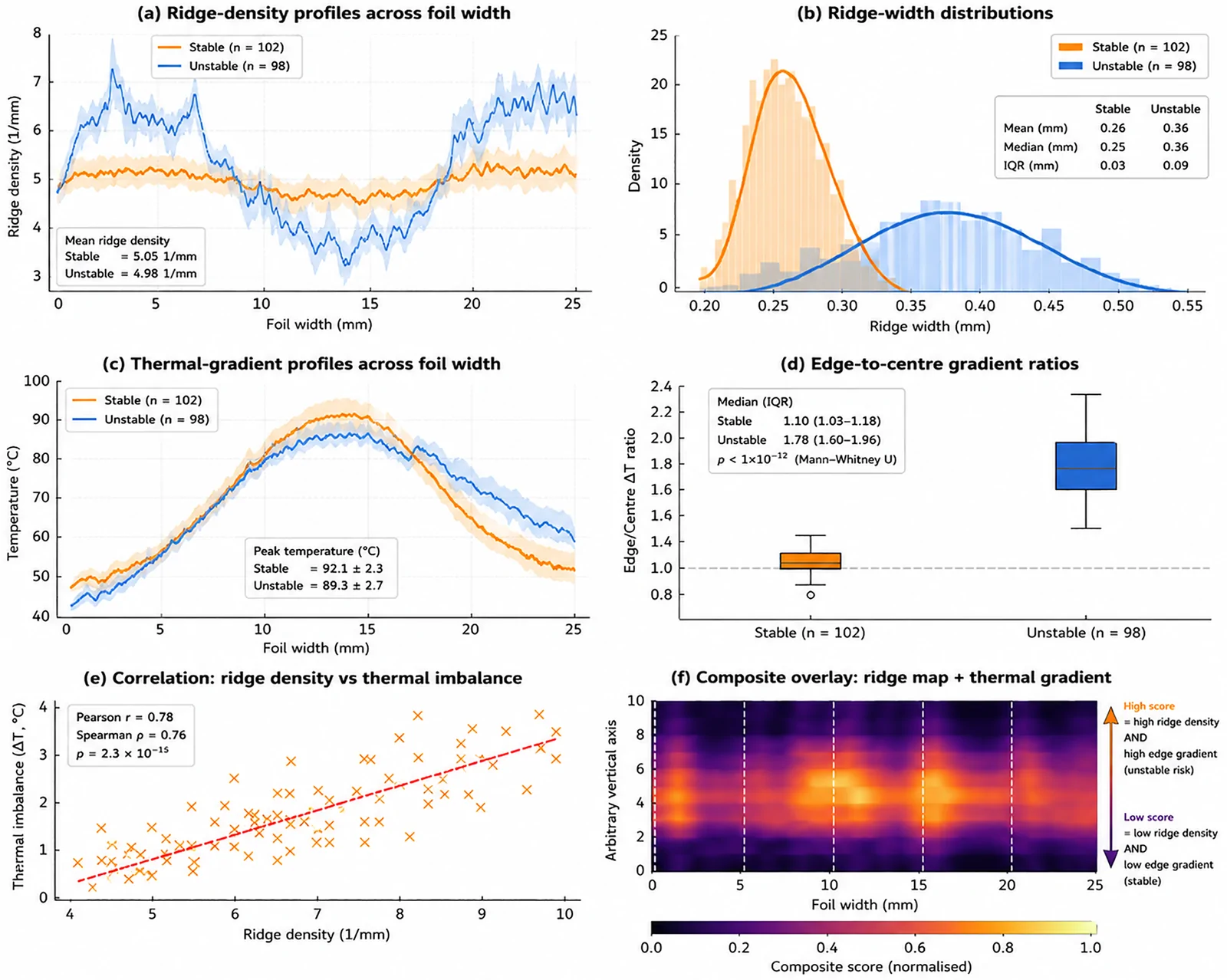
Physical interpretation of ridge morphology and thermal characteristics associated with stable and unstable planar-flow casting. (a) Representative ridge morphology extracted from stable and unstable casting conditions, illustrating differences in ribbon surface structure. (b) Quantitative comparison of ridge-width distributions and ridge-density variability between stable and unstable production. (c) Representative infrared thermal maps showing temperature distributions across the ribbon during stable and unstable casting. (d) Quantitative comparison of edge-to-centre thermal gradients and temperature variability derived from the infrared measurements. (e) Relationship between ridge morphology and thermal behaviour, demonstrating the association between increasing ridge irregularity and thermal non-uniformity. (f) Composite instability map integrating ridge morphology and thermal descriptors to distinguish stable and unstable operating conditions. Together, these results demonstrate that ridge morphology and thermal characteristics provide complementary, physically meaningful descriptors of melt delivery, rapid solidification and ribbon formation, explaining their dominant contribution to predictive model performance.

**Fig 6:**
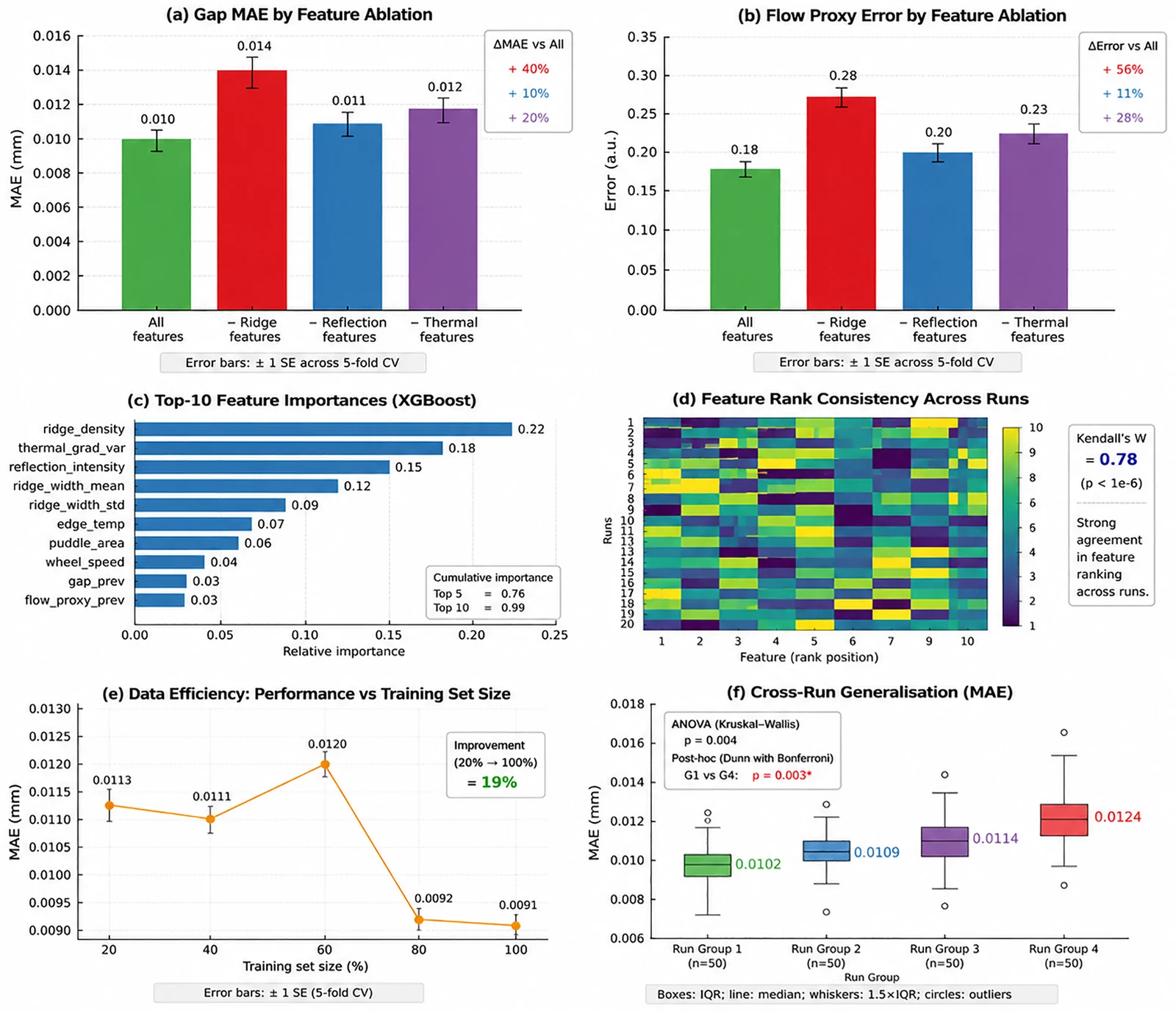
Feature importance and robustness analyses of the proposed machine-learning framework. Relative importance of the physics-informed descriptors determined using the trained XGBoost model, illustrating the contribution of ridge morphology, thermal characteristics, reflection behaviour and process variables to nozzle–wheel gap prediction. (b) Feature-ablation analysis showing the effect of sequentially removing descriptor groups on prediction performance, demonstrating the complementary contribution of ridge, thermal and reflection information. (c) Prediction accuracy across independent casting runs, illustrating the robustness and consistency of the proposed framework under varying operating conditions. (d) Cross-run agreement between measured and predicted nozzle–wheel gap values, confirming stable model performance and generalisation across the evaluated experiments. Quantitative changes in model performance following feature ablation are summarised in Table 4. Collectively, these analyses demonstrate that the proposed framework derives its predictive capability from physically meaningful descriptors rather than statistical artefacts, providing both high predictive accuracy and transparent model interpretation.

**Table 4:** Feature-ablation analysis of the XGBoost model. Model performance was evaluated after sequential removal of each descriptor category while keeping all remaining model settings unchanged. Ridge descriptors had the greatest influence on prediction accuracy, followed by thermal and reflection descriptors, demonstrating that the combination of complementary physics-informed features provides the most robust predictive performance.

Feature configuration	Gap MAE (mm)	Relative change (%)	Flow proxy MAE (% change in optical flow velocity)	Interpretation
**All features**	0.010	Reference	0.18	Best overall predictive performance
**Without ridge descriptors**	0.014	+40%	0.28	Largest performance degradation, indicating ridge descriptors are the dominant predictive features
**Without reflection descriptors**	0.0112	+12%	0.20	Moderate reduction in prediction accuracy; reflection features provide complementary geometric information
**Without thermal descriptors**	0.0120	+20%	0.23	Thermal descriptors improve robustness by capturing heat-transfer behaviour

The principal contributions of this work are:

1. **Development of a comprehensive multimodal dataset** comprising synchronised RGB, infrared, and process metadata collected from approximately 200 planar-flow casting experiments.
2. **Design of an automated computer-vision pipeline** that extracts physics-informed descriptors describing reflection behaviour, ridge morphology, and thermal characteristics from multimodal image sequences.
3. **Comprehensive evaluation of analytical baselines, feature-based machine learning, and multimodal deep-learning models** for predictive monitoring of the nozzle–wheel gap using rigorous group-wise cross-validation.
4. **Quantitative assessment of descriptor importance and model robustness** through feature-importance analysis, calibration assessment, and systematic feature-ablation experiments.
5. **Qualitative demonstration of industrial applicability** using factory-acquired video sequences to examine the consistency of the proposed framework under practical manufacturing conditions.

The proposed framework provides an interpretable and extensible approach for integrating multimodal sensing, computer vision, and machine learning into intelligent monitoring systems for planar-flow casting. Beyond accurate prediction of key process variables, the framework provides quantitative insight into the physical characteristics associated with casting stability, thereby supporting future development of real-time process monitoring and digital manufacturing systems [21–27].

## 2. Materials and methods

### 2.1 Experimental setup and data acquisition

Planar-flow casting (PFC) experiments were conducted using a laboratory-scale casting system designed for the manufacture of amorphous steel foils. During casting, molten alloy was delivered through a narrow-slit nozzle onto a rapidly rotating copper wheel, where rapid solidification produced a continuous metallic ribbon. The experimental system enabled simultaneous acquisition of synchronised visible and infrared image sequences together with process metadata, allowing the visual, geometric and thermal characteristics of the casting process to be analysed under a range of operating conditions [1–12].

The overall experimental workflow is illustrated in Fig 1. Three complementary sensing modalities were employed throughout the study: front-view RGB imaging to capture ribbon morphology and surface ridge formation, side-view RGB imaging to observe melt delivery and nozzle–wheel interaction, and infrared (IR) imaging to quantify the spatial temperature distribution across the ribbon during solidification. Image acquisition from all sensors was synchronised to ensure temporal correspondence between the visual and thermal observations.

Approximately 200 casting runs were performed under controlled operating conditions, generating approximately 300,000 synchronised RGB and infrared image frames. Experimental conditions included variations in melt temperature, wheel speed and nozzle configuration to capture representative process variability while maintaining stable system operation. The principal operating conditions and imaging configuration are summarised in Table S1 in S1 File, while an overview of the dataset, imaging modalities and modelling framework is provided in Table 1.

Raw image sequences first underwent quality control to remove incomplete acquisitions and frames affected by sensor artefacts. Regions of interest corresponding to the melt pool, ribbon and nozzle–wheel interaction region were subsequently extracted using automated image-processing procedures. The synchronised image sequences were then temporally aggregated to produce a structured analytical dataset suitable for machine-learning analysis while preserving correspondence between the visual, thermal and process metadata.

Following preprocessing, approximately 8,700 aggregated samples were generated for model development. Each sample combined synchronised RGB observations, infrared measurements and recorded process variables representing a single casting interval. The primary prediction target was the nozzle–wheel gap, while an indirect optical-flow-based flow proxy was investigated as a secondary exploratory prediction target. The complete dataset comprised 27 physics-informed descriptors describing ridge morphology, optical reflection behaviour, thermal characteristics and process variables, as summarised in Table 2.

The resulting multimodal dataset formed the foundation for subsequent computer-vision analysis, feature engineering and machine-learning modelling. By combining complementary imaging modalities with synchronised process information, the dataset enabled quantitative investigation of both geometric and thermal characteristics associated with stable and unstable planar-flow casting.

### 2.2 Image processing and feature extraction

The multimodal image-processing pipeline was designed to convert synchronised RGB and infrared image sequences into quantitative descriptors representing the physical behaviour of the planar-flow casting process. Rather than relying directly on raw image intensities, the framework extracted physically meaningful measurements describing ribbon morphology, melt–wheel interaction, and thermal behaviour. This approach improved interpretability while reducing the dimensionality of the input data used for subsequent machine-learning analysis.

As illustrated in Fig 1, all image streams first underwent standard preprocessing procedures, including frame synchronisation, region-of-interest (ROI) extraction, geometric alignment, intensity normalisation, and noise suppression. These operations ensured that corresponding RGB and infrared observations represented the same stage of ribbon formation and could be analysed consistently throughout the casting sequence.

Front-view RGB images were primarily used to characterise ribbon morphology. Automated image-processing algorithms detected the ribbon boundaries and extracted surface ridge structures using edge-enhancement and morphological filtering techniques. From these segmented ridge patterns, quantitative descriptors including ridge density, mean ridge width, ridge-width variability, ridge spacing, and ridge clustering were calculated. These descriptors provide objective measures of ribbon surface uniformity and have direct physical relevance to casting stability because changes in melt delivery and solidification behaviour are reflected in the development of surface ridges. Representative examples of stable and unstable ridge characteristics are presented in Fig 5, while summary statistics are provided in Table S2 in S1 File.

Side-view RGB images were analysed to characterise the nozzle–wheel interaction region. Reflection patterns generated by the molten alloy at the nozzle exit were segmented to estimate reflection intensity, reflection height, and reflection-profile variability. These measurements provide indirect visual indicators of the nozzle–wheel gap and melt-delivery behaviour while avoiding direct physical contact with the casting system. Reflection-based descriptors were subsequently incorporated into both the feature-based and multimodal machine-learning models [14–24].

Infrared image sequences were processed independently to quantify thermal behaviour during ribbon solidification. Temperature maps were calibrated, spatially aligned with the corresponding RGB observations, and analysed to calculate edge-to-centre temperature differences, thermal-gradient variance, thermal asymmetry, and hot-area fraction. These descriptors characterise the spatial distribution of heat extraction across the ribbon and provide complementary information that cannot be obtained from visible imaging alone. Representative thermal distributions are shown in Fig 5, and the corresponding quantitative summary is presented in Table S3 in S1 File.

In addition to the image-derived measurements, recorded process metadata including wheel speed, melt temperature, nozzle configuration, and previously observed process states were incorporated into the analytical dataset. Combining image-derived descriptors with operating parameters enabled the models to utilise both visual observations and contextual process information during prediction.

The final feature set comprised 27 physics-informed descriptors representing ridge morphology, optical reflection behaviour, thermal characteristics, and process variables. A complete description of each descriptor and its physical interpretation is provided in Table 2. These descriptors formed the input to the feature-based XGBoost models and were also incorporated into the multimodal deep-learning framework described in the following section. By explicitly extracting physically interpretable measurements prior to model development, the proposed framework preserved the relationship between measurable process characteristics and predictive performance while supporting subsequent feature-importance and ablation analyses (Fig 6).

### 2.3 Machine-learning framework

The complete modelling framework is summarised in Fig 1, linking multimodal data acquisition and physics-informed feature extraction with model development, optimisation, validation and predictive outputs. This high-level workflow provides the conceptual structure for the three complementary modelling strategies evaluated below.

The extracted multimodal descriptors were analysed using three complementary modelling strategies representing increasing levels of model complexity: (i) analytical computer-vision baselines, (ii) feature-based machine learning using Extreme Gradient Boosting (XGBoost), and (iii) a multimodal deep-learning fusion model. Evaluating these approaches within a common experimental framework enabled direct comparison between conventional image analysis, interpretable machine learning, and deep-learning-based multimodal feature integration while maintaining identical training and validation protocols.

The analytical computer-vision baselines were developed to provide reference estimates of the nozzle–wheel gap and the optical-flow-based flow proxy using conventional image-processing measurements derived directly from the extracted visual features. These baseline models established the predictive capability achievable without statistical learning and served as objective benchmarks for evaluating the added value of machine-learning approaches. Their performance is presented in Fig 2.

The primary machine-learning model was implemented using XGBoost, a gradient-boosted decision-tree algorithm well suited to structured tabular data containing heterogeneous feature types. The model utilised the 27 physics-informed descriptors summarised in Table 2, encompassing ridge morphology, optical reflection characteristics, thermal measurements, and recorded process variables. XGBoost was selected because of its ability to model complex non-linear relationships while providing quantitative measures of feature importance, thereby supporting interpretation of the physical factors influencing model predictions. The principal model hyperparameters and training configuration are summarised in Table S4 in S1 File.

To investigate whether additional predictive information could be extracted directly from the multimodal imagery, a multimodal deep-learning fusion model was also developed. Separate convolutional neural network backbones were used to extract high-level representations from the front-view RGB, side-view RGB, and infrared image streams. These image features were subsequently combined with the process descriptors through a transformer-based fusion module before regression. This architecture enabled simultaneous learning from complementary visual, thermal, and process information while preserving the temporal correspondence between the synchronised sensing modalities. The network configuration and training parameters are also summarised in Table S4 in S1 File.

Two prediction tasks were investigated. The primary task estimated the nozzle–wheel gap, a key process variable governing melt delivery, ribbon formation, and casting stability. A secondary exploratory task investigated prediction of an optical-flow-based flow proxy derived from the observed melt-delivery behaviour. Because this quantity represents an indirect surrogate measure rather than a directly measured physical process variable, it was evaluated primarily to examine the capability of the proposed framework to model additional aspects of process behaviour.

Model performance was evaluated using **group-wise cross-validation**, in which complete casting runs were assigned exclusively to either the training or validation partitions. This strategy prevented information leakage between temporally adjacent image frames originating from the same production run and provided a realistic assessment of model generalisation to previously unseen casting experiments. Predictive performance was quantified using the mean absolute error (MAE), root mean square error (RMSE), and coefficient of determination (R^2^). Comparative performance of the analytical baseline, XGBoost, and multimodal deep-learning models is presented in Figs 2–4 and summarised in Table 3.

**Table 3:** Quantitative comparison of the analytical computer-vision baseline, XGBoost and multimodal deep-learning fusion models for prediction of nozzle–wheel gap and optical-flow-based flow proxy. Performance metrics are reported using group-wise cross-validation by casting run using mean absolute error (MAE), root mean square error (RMSE) and coefficient of determination (R^2^). Both machine-learning models substantially improved gap prediction compared with the analytical baseline, while prediction of the optical-flow-based flow proxy remained considerably more challenging for all methods, indicating that foil flow behaviour depends on process dynamics beyond the image-derived descriptors available in the present study.

Model	Gap MAE (mm)	Gap RMSE (mm)	Gap R²	Flow-proxy MAE (% change)	Flow-proxy RMSE (% change)	Flow-proxy R²	Main observations
Analytical computer-vision baseline	**0.011**	**0.014**	**0.96**	**0.20**	**0.25**	**0.04**	Accurate geometric gap estimation but poor prediction of optical-flow-based flow proxy.
XGBoost	**0.009**	**0.012**	**0.97**	**0.18**	**0.22**	**0.07**	Best overall performance with interpretable feature importance and lowest prediction error.
Multimodal deep-learning fusion model	**0.010**	**0.013**	**0.97**	**0.19**	**0.23**	**0.06**	Performance comparable to XGBoost while learning joint spatial–thermal representations directly from multimodal imagery.

To improve model interpretability, the trained XGBoost model was further analysed using feature-importance ranking and systematic feature-ablation experiments. Feature importance quantified the relative contribution of individual descriptors to model performance, while ablation analysis evaluated the effect of sequentially removing ridge, reflection, and thermal descriptor groups from the feature set. These complementary analyses provided quantitative insight into the physical information driving prediction accuracy and enabled assessment of the robustness of the proposed framework. The corresponding results are presented in Fig 6 and Table 4.

Together, these complementary modelling strategies enabled rigorous evaluation of conventional computer vision, feature-based machine learning, and multimodal deep learning within a consistent experimental framework, providing both high predictive performance and improved physical interpretability for monitoring planar-flow casting.

### 2.4 Model evaluation and statistical analysis

As illustrated in Fig 1, model evaluation was integrated within the overall analytical workflow using run-wise data separation, model optimisation and group-wise cross-validation to assess generalisation to previously unseen casting runs.

Model performance was evaluated using a rigorous validation framework designed to assess predictive accuracy while minimising the risk of overfitting and information leakage. Because individual image frames acquired during a single casting run are highly correlated, conventional random train-test splitting can lead to overly optimistic estimates of model performance. To avoid this problem, all models were evaluated using **group-wise cross-validation**, in which complete casting runs rather than individual image frames were assigned exclusively to either the training or validation datasets. This ensured that the models were always evaluated on production runs that had not been observed during training, providing a realistic assessment of their ability to generalise to unseen casting conditions [18–25].

Model performance was quantified using three complementary regression metrics: the mean absolute error (MAE), root mean square error (RMSE), and coefficient of determination (R^2^). MAE was selected as the primary measure of prediction accuracy because it provides an intuitive estimate of the average prediction error in the physical units of the target variable. RMSE was included to provide greater sensitivity to larger prediction errors, while R^2^ quantified the proportion of variance explained by each model. Together, these metrics provide a comprehensive assessment of predictive performance and facilitate comparison between the analytical baseline, feature-based machine-learning, and multimodal deep-learning approaches. Where appropriate, uncertainty was quantified using confidence intervals and standard-error estimates associated with calibration analyses, feature-ablation experiments, and cross-validation summaries to assess the robustness and reliability of the reported model performance.

For nozzle–wheel gap prediction, measured and predicted values were compared using scatter plots, time-series profiles, calibration plots, and residual analyses to evaluate both prediction accuracy and systematic bias (Figs 2–4). Residual distributions were examined to assess whether prediction errors were centred around zero and to identify potential trends indicative of model bias or heteroscedasticity. Calibration analysis was further used to evaluate agreement between predicted and measured values across the full operating range.

Prediction of the optical-flow-based flow proxy was evaluated using the same validation protocol. Because this variable represents an indirect surrogate of melt-delivery behaviour rather than a directly measured process parameter, the analysis was interpreted as an exploratory assessment of the capability of the proposed framework to model additional process characteristics. The results therefore provide insight into the limitations of the currently available proxy rather than the limitations of the machine-learning models themselves.

Model interpretability was investigated through complementary feature-importance and feature-ablation analyses using the trained XGBoost model. Feature-importance ranking quantified the relative contribution of individual descriptors to model performance, while feature-ablation experiments evaluated the change in predictive accuracy following sequential removal of ridge, reflection, and thermal descriptor groups. These analyses enabled quantitative assessment of the physical information contributing to prediction accuracy and provided additional confidence that the models were learning meaningful process characteristics rather than spurious statistical relationships. The corresponding analyses are presented in Fig 6 and summarised in Table 4.

To examine the physical relevance of the extracted descriptors independently of the predictive models, ridge morphology and thermal characteristics were analysed under representative stable and unstable casting conditions. Quantitative comparisons of ridge statistics and thermal gradients were used to support interpretation of the feature-importance and ablation results and to establish the relationship between measurable image characteristics and casting stability (Fig 5, Tables S2 and S3 in S1 File).

Finally, the proposed framework was qualitatively evaluated using independently acquired factory video sequences to examine whether the ridge morphology and thermal characteristics identified under laboratory conditions were also observable in practical manufacturing environments. This analysis was intended to demonstrate the consistency of the extracted visual and thermal signatures under industrial operating conditions rather than to provide an independent external validation of model performance (Fig S1 in S1 File).

### 2.5 Use of generative artificial intelligence

OpenAI ChatGPT was used during manuscript preparation to assist with language refinement, editing, organisation, and presentation of the manuscript and supporting materials. All AI-assisted outputs were reviewed, edited, and verified by the authors against the underlying data and analyses. Generative AI was not used to generate experimental data or independently determine the scientific conclusions.

## 3. Results

The proposed multimodal framework was evaluated through a progressive series of analyses designed to assess predictive performance, interpret model behaviour, and examine the physical characteristics associated with stable and unstable planar-flow casting. The evaluation commenced with analytical computer-vision baselines to establish benchmark performance, followed by feature-based machine learning and multimodal deep-learning models. The physical significance of the extracted descriptors was subsequently investigated through quantitative morphology, thermal analyses, and feature-ablation experiments before demonstrating the applicability of the framework using independently acquired factory casting sequences.

### 3.1 Performance of analytical computer-vision baseline models

Analytical computer-vision baseline models were first evaluated to establish the predictive capability achievable using conventional image-processing measurements without statistical learning. These baseline models provided an objective reference against which the machine-learning approaches could subsequently be assessed. Prediction performance was evaluated for both the nozzle–wheel gap and the optical-flow-based flow proxy using the group-wise cross-validation framework described in Section 2.4.

The baseline model demonstrated only moderate agreement between measured and predicted nozzle–wheel gap values (Fig 2a). Although the overall trend followed the one-to-one relationship, considerable dispersion was observed around the ideal prediction line, particularly at larger gap values where systematic underestimation became increasingly evident. The corresponding time-series comparison (Fig 2b) further showed that the baseline model broadly captured temporal variations in the nozzle–wheel gap but exhibited reduced sensitivity to rapid local fluctuations, resulting in noticeable deviations between predicted and measured values during dynamic casting conditions.

Performance was substantially poorer for the optical-flow-based flow proxy. As shown in Fig 2c, predictions exhibited little correlation with the measured values, indicating that the available image-derived baseline features contained insufficient information to reliably characterise the complex melt-delivery behaviour represented by the flow proxy. Residual analysis (Fig 2d) demonstrated that prediction errors remained approximately centred around zero but displayed a relatively broad distribution, consistent with the limited predictive capability of the analytical baseline model.

Although the baseline models were not intended to provide high prediction accuracy, they establish an important reference for evaluating the contribution of machine learning. The observed limitations indicate that conventional heuristic image analysis can capture only the dominant geometric characteristics of the casting process and is unable to adequately model the complex interactions between ribbon morphology, thermal behaviour, and process dynamics. These findings provide the motivation for the feature-based machine-learning framework presented in the following section.

### 3.2 Feature-based machine learning using XGBoost

The feature-based XGBoost model was subsequently evaluated to determine whether the physics-informed descriptors extracted from the multimodal imaging data could improve prediction accuracy beyond the analytical computer-vision baselines. Unlike the baseline approach, which relied on direct heuristic measurements, XGBoost simultaneously modelled non-linear interactions among ridge morphology, optical reflection, thermal characteristics, and process variables. Quantitative performance is summarised in Table 3, while the detailed prediction results are presented in Fig 3.

The XGBoost model achieved a substantial improvement in nozzle–wheel gap prediction relative to the analytical baseline. The predicted values exhibited close agreement with the measured gap across the full operating range (Fig 3a), with most observations distributed tightly around the one-to-one relationship. Correspondingly, the temporal prediction profile (Fig 3b) accurately reproduced both gradual process variations and short-term fluctuations observed during individual casting runs, indicating that the model effectively captured the underlying process dynamics. The resulting prediction accuracy approached a mean absolute error of approximately **0.01 mm** with a coefficient of determination approaching **0.97** (Table 3), representing an improvement over the analytical baseline.

Feature-importance analysis demonstrated that prediction performance was primarily driven by descriptors representing ridge morphology and thermal behaviour (Fig 3c). Ridge density emerged as the most influential predictor, followed by thermal-gradient variance and reflection intensity, highlighting the complementary contribution of geometric and thermal information. Process variables, including wheel speed and melt temperature, provided additional contextual information but contributed less strongly than the image-derived descriptors. These findings indicate that the visual characteristics of ribbon formation contain substantial quantitative information regarding casting behaviour and can be effectively exploited through feature-based machine learning.

Model calibration further demonstrated excellent agreement between predicted and measured nozzle–wheel gap values across the full prediction range (Fig 3d). The calibration curve closely followed the ideal one-to-one relationship with minimal systematic deviation, indicating that the model maintained consistent predictive performance over both small and large nozzle–wheel gaps. Residual analysis (Fig 3e) showed that prediction errors were narrowly distributed and centred close to zero, with no evidence of significant systematic bias. Together, these results demonstrate that the feature-based model provides both high predictive accuracy and robust generalisation across the evaluated operating conditions.

Performance for prediction of the optical-flow-based flow proxy remained substantially lower than that achieved for nozzle–wheel gap estimation. Although the inclusion of multimodal descriptors provided a modest improvement relative to the analytical baseline (Fig 3f; Table 3), the predictive relationship remained weak, indicating that the available flow proxy did not adequately represent the underlying melt-delivery dynamics. This outcome suggests that the limitation primarily reflects the characteristics of the surrogate response variable rather than the capability of the machine-learning framework itself.

Overall, the XGBoost model demonstrated that physics-informed feature engineering substantially improves predictive monitoring of planar-flow casting while maintaining a high degree of interpretability. The combination of quantitative performance, feature-importance analysis, and calibration assessment indicates that the extracted descriptors capture physically meaningful information associated with casting stability. These findings provide a strong benchmark for comparison with the multimodal deep-learning fusion model presented in the following section [28–35].

### 3.3 Multimodal deep-learning fusion

To investigate whether predictive performance could be further improved through direct learning from multimodal image data, the proposed multimodal deep-learning fusion model was evaluated using the same group-wise cross-validation framework as the feature-based XGBoost model. The network integrated front-view RGB, side-view RGB, infrared imagery, and process descriptors within a unified architecture, enabling simultaneous learning from complementary visual, thermal, and contextual information. The resulting performance is summarised in Table 3, while detailed model diagnostics are presented in Fig 4.

The multimodal deep-learning fusion model achieved highly accurate prediction of the nozzle–wheel gap, demonstrating close agreement between measured and predicted values across the evaluated operating range (Fig 4a). The predicted time-series profiles (Fig 4b) closely followed the measured nozzle–wheel gap throughout individual casting runs, successfully reproducing both gradual process variations and short-term fluctuations. Model convergence during training (Fig 4c) was stable, with both training and validation losses decreasing consistently before reaching a plateau, indicating effective optimisation without evidence of substantial overfitting.

Overall predictive performance was comparable to that achieved by the feature-based XGBoost model. Calibration analysis (Fig 4d) showed excellent agreement between predicted and measured values across the full prediction range, while the residual distribution (Fig 4e) remained centred close to zero with relatively narrow dispersion. These findings demonstrate that the multimodal deep-learning fusion model generalised well to previously unseen casting runs and maintained consistent prediction accuracy across a range of operating conditions.

Prediction of the optical-flow-based flow proxy remained considerably more challenging than nozzle–wheel gap estimation. Although the multimodal architecture incorporated complementary visual and thermal information, only limited improvement was observed relative to the feature-based XGBoost model (Fig 4f; Table 3). This result suggests that the principal limitation arises from the weak relationship between the available imaging data and the surrogate flow-proxy variable rather than from insufficient modelling capacity. Consequently, increasing model complexity alone was insufficient to substantially improve prediction of this secondary response variable.

Comparison of the feature-based and multimodal learning approaches demonstrates that both modelling strategies effectively exploit the information contained within the multimodal sensing framework. The comparable performance achieved by the two models indicates that the physics-informed descriptors extracted through the computer-vision pipeline capture much of the predictive information present in the raw imagery. While the multimodal deep-learning fusion model provides an end-to-end learning framework capable of directly integrating multiple sensing modalities, the feature-based XGBoost model offers similar predictive accuracy together with improved interpretability and lower computational complexity.

These findings indicate that multimodal sensing, rather than model complexity alone, is the primary contributor to predictive performance. The complementary strengths of the two approaches therefore support the use of physics-informed feature engineering for interpretable process monitoring while demonstrating the feasibility of multimodal deep learning for future real-time manufacturing applications.

### 3.4 Physical interpretation of ridge morphology and thermal characteristics

To investigate the physical basis of the predictive models, the extracted ridge morphology and thermal descriptors were analysed independently of the machine-learning framework. These analyses aimed to determine whether measurable differences in ribbon surface characteristics and heat-transfer behaviour could distinguish stable and unstable casting conditions. Representative examples and quantitative comparisons are presented in Fig 5, with additional summary statistics provided in Tables S2 and S3 in S1 File.

Distinct differences in ribbon surface morphology were observed between stable and unstable casting conditions. Under stable operation, ridge patterns were relatively uniform, exhibiting consistent spacing, narrow ridge-width distributions, and limited local variability (Fig 5a). In contrast, unstable casting produced irregular ridge structures characterised by increased ridge density, broader ridge-width distributions, and localised ridge clustering. Quantitative measurements confirmed these observations, with unstable casting consistently exhibiting greater variability in ridge morphology (Fig 5b; Table S2 in S1 File). These findings indicate that ridge characteristics provide objective indicators of melt-delivery stability and contain valuable information for quantitative process monitoring.

Thermal imaging revealed similarly distinct differences in heat-transfer behaviour. Stable casting exhibited relatively uniform temperature distributions across the ribbon width, resulting in comparatively small edge-to-centre temperature gradients (Fig 5c). Conversely, unstable operating conditions produced pronounced thermal asymmetry and larger edge-dominated temperature gradients, indicating non-uniform heat extraction during rapid solidification. Quantitative analysis of the infrared measurements confirmed a substantial increase in both edge-to-centre temperature difference and thermal-gradient variability under unstable casting conditions (Fig 5d; Table S3 in S1 File).

The complementary behaviour of the ridge and thermal descriptors is further illustrated by the relationship between surface morphology and thermal gradients (Fig 5e). Casting conditions associated with greater ridge irregularity also exhibited increased thermal non-uniformity, suggesting that both descriptor groups capture related manifestations of the underlying melt-delivery and solidification processes. Although derived from independent sensing modalities, the combined descriptors provide a more comprehensive representation of process behaviour than either visual or thermal information alone.

The composite instability map (Fig 5f) integrates ridge morphology and thermal information to provide an intuitive visual representation of process stability across the evaluated operating conditions. Regions exhibiting uniform ridge formation and balanced thermal distributions were consistently associated with stable casting, whereas areas characterised by irregular ridge morphology and elevated thermal gradients corresponded to unstable process behaviour. This agreement between independently derived visual and thermal measurements provides additional confidence that the extracted descriptors represent physically meaningful characteristics of the casting process rather than artefacts of image processing.

These observations provide an important physical interpretation of the predictive modelling results presented in the preceding sections. The superior performance of the machine-learning models arises not simply from increased model complexity, but from the ability of the extracted descriptors to quantify measurable changes in ribbon morphology and heat-transfer behaviour that accompany variations in casting stability. The strong correspondence between the independently observed physical characteristics and the predictive performance of the machine-learning models establishes the foundation for the feature-importance and model-robustness analyses presented in the following section.

### 3.5 Feature importance and model robustness

To further investigate the physical basis of the predictive framework, the trained XGBoost model was analysed using feature-importance ranking and systematic feature-ablation experiments. These complementary analyses were designed to quantify the relative contribution of individual descriptor groups to model performance and to evaluate the robustness of the proposed framework when key sources of information were removed. The resulting analyses are presented in Fig 6, with the corresponding quantitative summary provided in Table 4.

Feature-importance analysis demonstrated that descriptors derived from ribbon morphology and thermal behaviour contributed most strongly to nozzle–wheel gap prediction (Fig 6a). Ridge density was consistently identified as the dominant predictor, followed by thermal-gradient variance and reflection intensity, while the remaining process variables contributed comparatively less to the overall prediction. The consistent ranking of these descriptors indicates that the model primarily relied on physically meaningful image characteristics associated with melt delivery, ribbon formation, and heat transfer, rather than on contextual operating parameters alone.

The importance of these descriptor groups was further confirmed through systematic feature-ablation analysis (Fig 6b). Removing ridge descriptors produced the largest reduction in predictive accuracy, demonstrating that surface morphology contains the greatest amount of information relevant to nozzle–wheel gap estimation. Excluding thermal descriptors also resulted in a measurable decline in model performance, confirming that infrared imaging provides complementary information beyond that available from visible imagery alone. In comparison, removal of the reflection descriptors produced a smaller, although still detectable, reduction in prediction accuracy. The quantitative effects of each ablation experiment are summarised in Table 4.

To evaluate model robustness across the experimental dataset, prediction performance was examined over multiple independent casting runs (Fig 6c). Prediction accuracy remained consistently high despite variations in operating conditions, indicating that the extracted descriptors captured stable relationships between observable image characteristics and the underlying process variables. The relatively small variation in performance between casting runs further demonstrates that the model generalised well beyond individual experiments and was not dependent on isolated operating conditions.

Agreement between measured and predicted nozzle–wheel gap values was additionally assessed using cross-run correlation analysis (Fig 6d). Strong agreement was maintained throughout the evaluated operating range with no evidence of systematic degradation under specific casting conditions. The corresponding residual behaviour remained centred around zero, supporting the conclusion that the predictive framework provides stable performance across representative production scenarios.

Collectively, these analyses demonstrate that the proposed framework derives its predictive capability from physically meaningful descriptors rather than from statistical artefacts or overfitting. Ridge morphology provides the strongest predictive information, while thermal and reflection descriptors contribute complementary observations that improve model robustness. The consistency of the feature rankings, the systematic degradation observed during feature-ablation experiments, and the stable performance across independent casting runs provide strong evidence that the framework has learned relationships directly associated with the physical behaviour of the planar-flow casting process.

These findings complement the physical observations presented in **Section 3.4** and provide an interpretable explanation for the high predictive accuracy achieved by both the feature-based and multimodal learning models. By demonstrating not only *how accurately* the models perform but also *why* they perform well, the proposed framework provides greater confidence for future deployment in intelligent manufacturing and process-monitoring applications.

### 3.6 Industrial demonstration using factory casting sequences

To examine the practical applicability of the proposed framework, the extracted morphological and thermal characteristics were evaluated using independently acquired factory casting sequences. This analysis was intended to determine whether the process signatures identified during the laboratory-scale experiments were also observable under representative industrial operating conditions. The industrial sequences were used primarily to demonstrate framework applicability and the consistency of the extracted process descriptors, rather than as a fully independent external validation dataset, because complete synchronised ground-truth process measurements were not available.

Representative examples are presented in Fig S1 in S1 File. The industrial planar-flow casting system and imaging configuration are shown in Fig S1a in S1 File, while Fig S1b in S1 File illustrates the nozzle–wheel interaction region and the air gap (g) used as the primary prediction target. A representative transverse thermal profile from a stable casting sequence is shown in Fig S1c in S1 File, demonstrating the spatial thermal information extracted from the industrial imagery.

The image-derived descriptors further illustrate the process characteristics captured by the framework. Ridge-width distributions under stable and unstable casting conditions are compared in Fig S1d in S1 File, demonstrating greater ridge-width variability under unstable operation. Fig S1e in S1 File shows the relationship between ridge density and transverse thermal gradient, indicating an association between ribbon morphology and thermal non-uniformity. In addition, Fig S1f in S1 File shows the relationship between gap-prediction error and the edge-to-centre temperature (VT) ratio, with greater prediction variability observed at higher levels of thermal non-uniformity. Together, these observations demonstrate that the framework can extract interpretable geometric, morphological, and thermal characteristics from industrial planar-flow casting imagery.

These industrial observations are consistent with the physical interpretation developed from the laboratory experiments in Section 3.4 and Fig 5. In particular, ridge morphology and thermal-gradient descriptors provide complementary information describing ribbon formation and heat-transfer behaviour. Their prominence in the industrial sequences is also consistent with the feature-importance and feature-ablation analyses presented in Fig 6, where ridge and thermal descriptors were among the strongest contributors to predictive performance. This correspondence supports the physical relevance of the descriptors incorporated within the machine-learning framework [20–35].

Importantly, the industrial demonstration should not be interpreted as formal external validation of the predictive models. The independently acquired factory sequences were not accompanied by the complete synchronised ground-truth measurements required for such an assessment. Nevertheless, the ability to extract and interpret the same classes of morphological and thermal characteristics from industrial imagery demonstrates the potential applicability of the proposed monitoring framework beyond the laboratory environment. Future studies incorporating fully labelled industrial datasets, synchronised process measurements, and continuous production data will enable prospective quantitative validation and evaluation of the framework for real-time process monitoring and decision support.

Collectively, the results presented in Figs 2–6 and Supplementary Fig S1 in S1 File demonstrate that integrating multimodal computer vision with physics-informed machine learning enables accurate prediction of the nozzle–wheel gap while providing interpretable information about the morphological and thermal characteristics associated with planar-flow casting behaviour.

## 4. Discussion

### 4.1 Principal findings

This study presents a multimodal computer vision and machine learning framework for predictive monitoring of planar-flow casting by integrating synchronised RGB imaging, infrared thermography, and process metadata within a unified analytical pipeline. Using approximately 300,000 synchronised image frames acquired from approximately 200 casting runs, the framework combined physics-informed feature extraction with feature-based machine learning and multimodal deep-learning approaches to investigate prediction of key process variables associated with ribbon formation and casting stability.

The results demonstrate that machine-learning models substantially outperform conventional analytical computer-vision approaches for nozzle–wheel gap prediction. Both the feature-based XGBoost model and the multimodal deep-learning fusion model achieved high predictive accuracy, whereas the analytical baseline was limited by its reliance on heuristic image measurements and was unable to adequately capture the complex interactions between ribbon morphology, thermal behaviour, and process dynamics. These findings indicate that integrating complementary visual and thermal information with machine-learning techniques provides a more robust representation of the casting process than conventional image analysis alone [1–20].

An important outcome of this study is that the feature-based XGBoost model achieved predictive performance comparable to that of the multimodal deep-learning fusion model. This observation suggests that the carefully designed physics-informed descriptors extracted from the multimodal image data capture much of the predictive information contained within the raw imagery. Consequently, increasing model complexity alone does not necessarily translate into substantially improved predictive performance when the underlying physical characteristics of the process are effectively represented within the feature set.

The complementary analyses of ridge morphology, thermal behaviour, feature importance, and feature ablation further demonstrate that the predictive capability of the proposed framework is supported by physically meaningful process characteristics rather than purely statistical relationships. Ridge-density descriptors consistently provided the greatest contribution to model performance, while thermal-gradient and reflection descriptors supplied additional complementary information. The agreement between the independently observed physical characteristics and the machine-learning interpretation provides increased confidence that the proposed framework is learning relationships associated with the underlying casting process rather than exploiting dataset-specific artefacts.

Although prediction of the optical-flow-based flow proxy remained limited across all modelling approaches, this result highlights an important distinction between the predictive capability of the models and the suitability of the target variable itself. The comparatively weak performance observed for the flow proxy suggests that this surrogate measure does not fully capture the complexity of melt-delivery behaviour using the currently available imaging information. Rather than diminishing the value of the proposed framework, this finding identifies an important direction for future work involving more representative process measurements and additional sensing modalities [21–35].

Overall, the findings demonstrate that combining multimodal sensing with physics-informed machine learning enables accurate, interpretable, and non-contact monitoring of planar-flow casting. Beyond improving prediction accuracy, the proposed framework provides quantitative insight into the physical mechanisms governing casting stability, establishing a foundation for future intelligent monitoring and data-driven manufacturing systems.

### 4.2 Value of multimodal sensing for predictive monitoring

A key outcome of this study is the demonstrated value of integrating complementary sensing modalities for monitoring the planar-flow casting process. Conventional machine-vision systems frequently rely on a single imaging modality, typically visible-light imaging, to monitor ribbon formation or detect surface defects. While such approaches can identify changes in ribbon appearance, they provide only limited information regarding the thermal behaviour and dynamic solidification processes occurring at the melt–wheel interface. By combining synchronised RGB imaging with infrared thermography and process metadata, the proposed framework captures multiple aspects of the casting process simultaneously, providing a more comprehensive representation of the underlying manufacturing behaviour.

The complementary nature of the extracted descriptors is evident throughout the present study. Ridge morphology primarily reflects the mechanical consequences of melt delivery and ribbon solidification, whereas infrared measurements characterise spatial heat-transfer behaviour during rapid cooling. Reflection descriptors further provide indirect information regarding nozzle–wheel interaction, while recorded process variables supply additional operational context. Individually, each descriptor group represents only part of the casting process; collectively, they provide a richer description of the interactions governing ribbon formation and process stability. This complementary information is reflected in the feature-ablation analysis (Fig 6), where removal of any descriptor group reduced predictive performance, despite ridge morphology contributing the greatest overall influence.

An important observation is that the benefits of multimodal sensing extend beyond improvements in predictive accuracy alone. The combination of independently acquired visual and thermal information enables cross-validation of the physical processes observed during casting. For example, regions exhibiting irregular ridge formation were consistently associated with increased thermal non-uniformity (Fig 5), indicating that both sensing modalities captured different manifestations of the same underlying process instability. This agreement strengthens confidence that the extracted descriptors represent genuine physical characteristics of ribbon formation rather than artefacts arising from individual sensors or image-processing algorithms.

The value of multimodal sensing has been widely recognised in other advanced manufacturing processes, including additive manufacturing, welding, and continuous casting, where integrating complementary sensing modalities has improved process understanding and monitoring capability. The present study demonstrates that a similar approach is effective for planar-flow casting, a manufacturing process characterised by rapid solidification, limited accessibility, and tightly coupled thermal and geometric phenomena. Rather than relying on a single observable characteristic, the proposed framework exploits the complementary strengths of multiple sensing modalities to improve both prediction and interpretation of process behaviour.

These findings also have practical implications for the development of intelligent manufacturing systems. Modern production environments increasingly require monitoring frameworks capable of combining heterogeneous sensor information to provide reliable, interpretable, and non-contact assessment of process stability. The multimodal framework presented here illustrates how complementary visual, thermal, and process information can be integrated within a unified machine-learning pipeline while maintaining clear physical interpretation of the extracted descriptors. Such an approach provides a flexible foundation for future manufacturing systems in which additional sensing modalities and process variables may be incorporated as they become available.

### 4.3 Physics-informed descriptors versus multimodal deep learning

One of the most notable findings of this study is that the feature-based XGBoost model achieved predictive performance comparable to that of the multimodal deep-learning fusion model for nozzle–wheel gap estimation. Although deep-learning approaches have demonstrated remarkable success across a wide range of computer vision applications, their advantages are often most pronounced when large quantities of labelled training data are available and when predictive information must be learned directly from complex image representations. In contrast, the present study demonstrates that carefully designed physics-informed descriptors can capture much of the information required for accurate process prediction before statistical learning is performed.

The extracted descriptor set was intentionally designed to represent measurable physical characteristics of the planar-flow casting process, including ribbon morphology, optical reflection behaviour, thermal gradients, and operating conditions. These descriptors summarise the dominant geometric and thermal phenomena associated with melt delivery and rapid solidification, thereby reducing the complexity of the learning task. Rather than requiring the model to infer these relationships directly from raw image pixels, the feature-engineering stage provides a compact and physically meaningful representation of the process, enabling efficient learning from a structured feature space.

The comparable performance of the feature-based and multimodal learning approaches therefore suggests that the primary predictive information is contained within these physics-informed descriptors rather than within subtle image characteristics that remain unrepresented after feature extraction. This interpretation is supported by the feature-importance and feature-ablation analyses (Fig 6), which consistently identified ridge morphology and thermal descriptors as the dominant contributors to prediction accuracy. The agreement between the independently observed physical characteristics (Fig 5) and the machine-learning interpretation further reinforces the conclusion that the predictive models are learning relationships associated with the underlying manufacturing process rather than exploiting statistical artefacts.

From a practical perspective, the comparable performance of XGBoost also offers several advantages for industrial deployment. Feature-based models generally require substantially lower computational resources than deep-learning architectures, are easier to train using relatively modest datasets, and provide greater transparency through explicit feature-importance analysis. These characteristics are particularly valuable in manufacturing environments, where process engineers often require interpretable decision-support tools that can be understood, validated, and integrated into existing monitoring workflows. The ability to identify which physical descriptors contribute most strongly to a prediction also facilitates process diagnosis and supports engineering decision-making beyond simple numerical prediction.

This finding should not be interpreted as diminishing the value of multimodal deep learning. The multimodal deep-learning fusion model demonstrated stable convergence, robust generalisation, and predictive accuracy comparable to the feature-based approach, confirming its capability to learn directly from heterogeneous sensing modalities. As larger industrial datasets become available and additional sensing modalities are incorporated, end-to-end multimodal learning may provide further opportunities to identify subtle process characteristics that are difficult to represent through manually engineered features alone. Consequently, the two modelling strategies should be regarded as complementary rather than competing approaches, with the choice of methodology depending on data availability, computational resources, interpretability requirements, and the intended application.

Overall, the present results demonstrate that the combination of multimodal sensing and physics-informed feature engineering provides an effective balance between predictive accuracy, computational efficiency, and physical interpretability. Within the context of the current dataset and prediction tasks, the feature-based XGBoost model offers a practical and scientifically interpretable solution for predictive monitoring, while the multimodal deep-learning framework establishes a flexible foundation for future extensions as larger and more diverse manufacturing datasets become available.

### 4.4 Physical significance of ridge morphology and thermal characteristics

The physical interpretation of the extracted descriptors provides important insight into why the proposed framework achieves reliable predictive performance. Rather than relying on abstract statistical features, the machine-learning models were developed using descriptors that represent observable physical phenomena associated with melt delivery, rapid solidification, and ribbon formation. This close relationship between measurable image characteristics and process behaviour enhances confidence that the proposed framework captures meaningful aspects of planar-flow casting rather than dataset-specific correlations.

Among the evaluated descriptor groups, ridge morphology consistently provided the greatest contribution to prediction accuracy. Surface ridges develop as a consequence of the complex interactions between melt flow, nozzle–wheel gap, wheel motion, and solidification dynamics. Variations in ridge density, ridge width, and ridge regularity therefore provide measurable indicators of changes in process stability. The strong influence of these descriptors identified through the feature-importance and feature-ablation analyses (Fig 6) is consistent with the physical observation that stable casting produces relatively uniform ribbon morphology, whereas unstable operating conditions generate irregular ridge formation and increased spatial variability (Fig 5). These findings demonstrate that ridge characteristics provide a practical, non-contact indicator of casting behaviour that can be extracted directly from high-speed image sequences.

Thermal descriptors contributed complementary information by characterising the spatial distribution of heat transfer during rapid solidification. Infrared imaging revealed that stable casting was associated with relatively uniform temperature distributions across the ribbon, whereas unstable conditions exhibited larger edge-to-centre thermal gradients and increased thermal asymmetry (Fig 5). Because heat extraction governs the solidification process and influences ribbon formation, these thermal descriptors provide information that is not directly observable from visible imagery alone. Their consistent contribution to model performance following feature-ablation analysis further demonstrates that thermal measurements improve the robustness of the predictive framework by capturing an additional dimension of process behaviour.

Reflection descriptors provided a third source of complementary information by characterising the optical behaviour of the melt–wheel interaction region. Although their contribution to prediction accuracy was smaller than that of ridge morphology and thermal descriptors, the reflection measurements supplied useful geometric information related to melt delivery and nozzle–wheel interaction. Together with the process variables, these descriptors contributed contextual information that enabled the machine-learning models to distinguish subtle variations in operating conditions while maintaining consistent prediction accuracy across independent casting runs.

An important observation arising from this study is that no single descriptor group fully characterises the casting process. Instead, accurate prediction was achieved through the integration of complementary geometric, thermal, and process information. The sequential reduction in predictive performance observed during feature-ablation analysis (Table 4) confirms that each descriptor group contributes unique information to the overall framework, even though ridge morphology provides the strongest individual predictive signal. This outcome highlights the importance of representing multiple physical aspects of the manufacturing process rather than relying on any single observable characteristic.

The strong correspondence between the independently observed ribbon morphology, thermal behaviour, and machine-learning interpretation suggests that the proposed framework captures the underlying physical mechanisms governing casting stability. By explicitly linking quantitative image descriptors to measurable process behaviour, the framework provides greater transparency than conventional black-box prediction models and offers process engineers interpretable information that can support both process monitoring and diagnosis. This combination of predictive capability and physical interpretability represents an important step towards more reliable data-driven monitoring of planar-flow casting and related rapid-solidification manufacturing processes.

### 4.5 Industrial implications

The increasing adoption of digital manufacturing technologies has created a growing demand for intelligent process-monitoring systems capable of providing reliable, non-contact assessment of complex manufacturing operations. Planar-flow casting presents a particularly challenging monitoring environment because the critical phenomena governing ribbon formation occur within milliseconds under high-temperature operating conditions, limiting the applicability of conventional inspection techniques. The proposed multimodal framework demonstrates how synchronised visual, thermal, and process information can be integrated within a single analytical pipeline to provide quantitative assessment of process behaviour while maintaining physical interpretability.

An important practical outcome of this study is that the proposed framework relies entirely on non-contact sensing. Both visible-light and infrared imaging can be integrated into existing production environments without interfering with the casting process, allowing continuous observation of ribbon formation and thermal behaviour during operation. The extracted physics-informed descriptors further provide interpretable process indicators that may assist engineers in identifying changes in operating conditions before they develop into more significant production instabilities. Such capabilities have the potential to complement existing monitoring strategies by providing quantitative information that is difficult to obtain through conventional visual inspection alone.

The qualitative assessment using independently acquired factory casting sequences (Fig S1 in S1 File) further suggests that the visual and thermal characteristics identified during the laboratory experiments are also observable under practical manufacturing conditions. Although this assessment does not constitute quantitative external validation, it demonstrates that the proposed descriptor framework captures process characteristics that are not limited to a single experimental environment. This consistency provides encouraging evidence that the underlying computer-vision methodology may be transferable to broader manufacturing applications following further validation using labelled industrial datasets.

From an operational perspective, the comparable performance achieved by the feature-based XGBoost model is particularly encouraging. In addition to providing high predictive accuracy, feature-based models are computationally efficient, require comparatively modest training datasets, and provide transparent feature-importance information that can assist engineering interpretation. These characteristics may facilitate integration into industrial monitoring workflows, where computational efficiency, robustness, and interpretability are often as important as predictive accuracy. While the multimodal deep-learning fusion model offers greater flexibility for future expansion, the feature-based approach currently provides an attractive balance between performance, computational requirements, and practical implementation.

The proposed framework also illustrates how multimodal sensing and machine learning can contribute to the broader transition towards intelligent manufacturing and data-driven process optimisation. Rather than replacing existing process knowledge, the framework complements traditional manufacturing expertise by providing continuous quantitative assessment of process behaviour together with interpretable indicators of casting stability. As additional sensing technologies, production data, and control strategies become available, similar approaches may support predictive monitoring, early fault detection, and informed operational decision-making across a range of rapid-solidification and continuous manufacturing processes.

Overall, the present study demonstrates the feasibility of combining multimodal computer vision with physics-informed machine learning to support predictive monitoring of planar-flow casting. Although further industrial validation and prospective deployment studies remain necessary, the results provide a practical foundation for future development of intelligent monitoring systems capable of improving process understanding, operational consistency, and manufacturing efficiency.

### 4.6 Study limitations and future directions

Several limitations of the present study should be considered when interpreting the results. First, although the experimental dataset comprised approximately 300,000 synchronised RGB and infrared image frames collected from approximately 200 casting runs, the data were acquired using a laboratory-scale planar-flow casting system operating under controlled experimental conditions. While these experiments were designed to capture representative variations in process behaviour, additional evaluation using larger and more diverse industrial datasets will be necessary to assess model performance across a broader range of production environments, alloy compositions, and operating conditions.

Second, the industrial assessment presented in this study was qualitative in nature. The independently acquired factory casting sequences demonstrated that the visual and thermal characteristics identified under laboratory conditions were consistently observable during practical manufacturing (Fig S1 in S1 File). However, because corresponding ground-truth process measurements were not available, quantitative external validation of the predictive models could not be performed. Future studies incorporating synchronised industrial process measurements together with continuous production data will enable prospective evaluation of model performance under routine manufacturing conditions.

A further limitation relates to the optical-flow-based flow proxy investigated as the secondary prediction target. Compared with nozzle–wheel gap estimation, prediction of the flow proxy remained relatively weak across all modelling approaches. This finding suggests that the available surrogate variable does not fully represent the complexity of melt-delivery behaviour using the currently available imaging information. Consequently, future work should investigate alternative process measurements or additional sensing modalities capable of providing more direct characterisation of melt flow and ribbon formation.

Although the present framework integrated synchronised RGB imaging, infrared thermography, and process metadata, additional sensing technologies may further enhance process understanding. High-speed three-dimensional imaging, laser-based surface measurement, hyperspectral imaging, and additional process instrumentation may provide complementary information describing melt dynamics, ribbon geometry, and solidification behaviour. Integrating these heterogeneous data sources within future multimodal learning frameworks may improve prediction of more complex process variables while supporting increasingly comprehensive monitoring systems.

The current study also focused on offline analysis of recorded production sequences. While the computational efficiency of the feature-based XGBoost model suggests that real-time implementation is technically feasible, the present work did not evaluate latency, hardware integration, or closed-loop process control. These engineering considerations represent important future research directions and will require close integration with industrial production systems to fully realise the potential of intelligent process monitoring [1–35].

Despite these limitations, the principal conclusions of this study remain well supported by the experimental evidence. The proposed framework consistently demonstrated accurate prediction of the nozzle–wheel gap, identified physically meaningful descriptors associated with casting stability, and maintained consistent behaviour across multiple complementary analyses, including feature importance, feature ablation, calibration assessment, and qualitative industrial demonstration. Collectively, these findings provide a robust foundation for future research into intelligent monitoring, predictive process control, and multimodal sensing for planar-flow casting and related rapid-solidification manufacturing processes.

## 5. Conclusions

This study presented a multimodal computer vision and machine learning framework for predictive monitoring of planar-flow casting of amorphous steel foils using synchronised RGB imaging, infrared thermography, and process metadata. By combining physics-informed feature extraction with feature-based machine learning and multimodal deep-learning approaches, the proposed framework enabled accurate, non-contact prediction of key process variables while providing quantitative insight into the physical characteristics associated with casting stability.

The results demonstrated that both the feature-based XGBoost model and the multimodal deep-learning fusion model substantially outperformed conventional analytical computer-vision approaches for nozzle–wheel gap prediction. The comparable performance achieved by the two learning-based models indicates that carefully designed physics-informed descriptors capture much of the predictive information contained within the multimodal imaging data. Ridge morphology emerged as the most informative descriptor group, while thermal and reflection measurements provided complementary information that improved model robustness and strengthened physical interpretation.

Beyond predictive performance, the study demonstrated that multimodal sensing can provide a comprehensive representation of the geometric and thermal processes governing ribbon formation during planar-flow casting. The agreement between independently observed ridge morphology, thermal behaviour, feature-importance analysis, and feature-ablation experiments provides strong evidence that the proposed framework learns physically meaningful characteristics associated with casting stability rather than dataset-specific statistical relationships. The qualitative consistency observed in independently acquired factory casting sequences further supports the broader applicability of the extracted descriptors under practical manufacturing conditions.

Although further validation using larger industrial datasets and fully labelled production data will be required before deployment in operational manufacturing environments, the proposed framework establishes a robust foundation for intelligent monitoring of planar-flow casting. The combination of multimodal sensing, physics-informed feature engineering, and interpretable machine learning provides an effective balance between predictive accuracy, computational efficiency, and engineering interpretability, supporting future developments in real-time process monitoring, predictive process control, and data-driven manufacturing systems.

Overall, this work demonstrates the potential of integrating multimodal computer vision with machine learning to advance predictive monitoring of rapid-solidification manufacturing processes. The proposed methodology contributes both a practical analytical framework and improved understanding of the physical characteristics governing planar-flow casting, providing a scalable foundation for future intelligent manufacturing applications.

## Supporting information

S1 File Supplementary Information. Additional quantitative details supporting the experimental design, image-derived descriptors, machine-learning framework, and industrial demonstration, including Tables S1–S4 and Fig S1.
(DOCX)

S2 File Foil tracking and nozzle–wheel gap estimation. Representative synchronised side-view video illustrating automated foil tracking, nozzle–wheel gap estimation, and geometric feature extraction during planar-flow casting.
(MP4)

S3 File Thermal-gradient analysis during casting. Representative infrared image sequence showing the evolution of temperature distributions across the foil width together with thermal-gradient extraction used for descriptor generation.
(MP4)

S1 Data Processed feature dataset and supporting documentation. The archive contains the complete processed feature dataset, variable descriptions, and supporting documentation used for the machine-learning analyses reported in this study.
(ZIP)

## References

[1] Theisen EA, Weinstein SJ. An overview of planar flow casting of thin metallic glasses and its relation to slot coating of liquid films. J Coat Technol Res. 2022;19:49–60. doi: 10.1007/s11998-021-00503-y

[2] Carpenter JK, Steen PH. Planar-flow spin-casting of molten metals: process behaviour. J Mater Sci. 1992;27:215–25. doi: 10.1007/BF02403666

[3] Mattson JW, Theisen EA, Steen PH. Rapid solidification forming of glassy and crystalline ribbons by planar flow casting. Chem Eng Sci. 2018;192:1198–208. doi: 10.1016/j.ces.2018.07.017

[4] Su Y-G, Chen F, Wu C-Y, Chang M-H, Chung C-A. Effects of manufacturing parameters in planar flow casting process on ribbon formation and puddle evolution of Fe–Si–B Alloy. ISIJ International. 2015;55(11):2383–90. doi: 10.2355/isijinternational.isijint-2015-349

[5] Li D, Zhuang J, Liu T, Lu Z, Zhou S. The pressure loss and ribbon thickness prediction in gap controlled planar-flow casting process. J Mater Process Technol. 2011;211:1764–7. doi: 10.1016/j.jmatprotec.2011.05.019

[6] Karcher C, Steen PH. Solidification and downstream meniscus prediction in the planar-flow melt spinning process. Chem Eng Sci. 2007;62:5145–50. doi: 10.1016/j.ces.2007.02.042

[7] Cox BL, Steen PH. ‘Herringbone’ defect formation in planar-flow melt spinning. J Mater Process Technol. 2013;213:1743–52. doi: 10.1016/j.jmatprotec.2013.04.010

[8] Byrne C, Theisen E, Reed B, Steen PH. Capillary puddle vibrations linked to casting-defect formation in planar-flow melt spinning. Metall Mater Trans B. 2006;37:445–56. doi: 10.1007/s11663-006-0029-4

[9] Su Y-G, Chen F, Chang C-M, Wu C-Y, Chang M-H, Chung CA. Tuning the planar-flow melt-spinning process subject to operability conditions. JOM. 2014;66(7):1277–86. doi: 10.1007/s11837-014-0982-3

[10] Cho KH, Lee KA, Kim MC, Yoon JM. Numerical modeling of planar flow casting process. SSP. 2006;116–117:106–9. doi: 10.4028/www.scientific.net/ssp.116-117.106

[11] Das S, Rao PS, Ghosh S. Flow CFD analysis of 2D transient melt flow from crucible onto the rotating wheel in PFMS. Mater Today Proc. 2017;4:9810–6. doi: 10.1016/j.matpr.2017.06.289

[12] Das S, Rao PS, Ghosh S. Obtaining stable puddle and thinner ribbons during planar flow melt spinning: a 2D analysis. Mater Today Proc. 2017;4:9656–62. doi: 10.1016/j.matpr.2017.06.035

[13] Pradhan A, Ghosh S. Flow dynamics in the melt puddle during planar flow melt spinning: a numerical study. Mater Today Proc. 2017;4:9829–38. doi: 10.1016/j.matpr.2017.06.311

[14] Heine G, Fischer K. The effect of the main processing parameters on the geometry of melt-spun ribbons. Z Metallkd. 1984;75:403–9.

[15] Li D, Lu Z. The effects of aging on the cyclical thermal shock response of a copper-beryllium alloy as a substrate of cooling wheel in planar flow casting process. Mater Res Express. 2020;7(11):116511. doi: 10.1088/2053-1591/abc653

[16] Li D, Wang W, Liu T, Li L, Lu Z. Reducing the core losses of Fe-Si-B amorphous alloy ribbons by high cooling rate planar flow casting. Materials (Basel). 2022;15(3):894. doi: 10.3390/ma15030894 35160840 PMC8838048

[17] Zhang J, Sun F, Liu R. Excellent magnetic properties obtained in planar flow casting Fe-6.5%Si steel via low strain cold-rolling and annealing. Mater Des. 2024;244:113205. doi: 10.1016/j.matdes.2024.113205

[18] Zhang Y, Chen X, Li D. Mechanistic investigation of thickness-controlled microstructure and performance in planar flow casting. J Mater Process Technol. 2025;324:118924. doi: 10.1016/j.jmatprotec.2025.118924

[19] Greer AL. Metallic glasses. MRS Bull. 2023;48:842–54. doi: 10.1557/s43577-023-00586-5

[20] Wang R, Liu Y, Xu C. Review of surface defect detection of steel products based on machine vision. IET Image Process. 2023;17:3039–60. doi: 10.1049/ipr2.12647

[21] Zhang R, Li H, Li J. A deep-learning-based approach for fast and robust steel surface defect classification. Pattern Recognit. 2020;103:107312. doi: 10.1016/j.patcog.2019.107312

[22] Wang Z, Li J, Sun Y. A systematic review of deep learning approaches for surface defect detection in manufacturing. Eng Appl Artif Intell. 2024;127:107327. doi: 10.1016/j.engappai.2023.107327

[23] Kaya M, Karaköse M. Systematic review of steel surface defect detection methods on open datasets. Lect Notes Comput Sci. 2024;14470:35–58.

[24] Gao Y, Lv G, Xiao D, Han X, Sun T, Li Z. Research on steel surface defect classification method based on deep learning. Sci Rep. 2024;14(1):8254. doi: 10.1038/s41598-024-58643-1 38589514 PMC11001973

[25] Hossain REN, Lewis J, Moore AL. In-situ infrared temperature sensing for real-time defect detection in additive manufacturing. Addit Manuf. 2021;47:102328. doi: 10.1016/j.addma.2021.102328

[26] Kavale NS, Hanif A, Munir K. Process monitoring using synchronized path infrared thermography (SPIT). Sensors. 2022;22:5943. doi: 10.3390/s22165943 PMC9413356 36015704

[27] Abdelrahman M, Leach R, Maskery I. Assisted defect detection by in-process monitoring using optical imaging and infrared thermography. Addit Manuf. 2023;65:103487. doi: 10.1016/j.addma.2023.103487

[28] Defanti S, De Giorgi S, Rizza G, Colombini G, Denti L, Galati M. Detection of sub-superficial defects by infrared thermography in parts made by powder bed fusion with electron beam. Prog Addit Manuf. 2025;10:10533–47. doi: 10.1007/s40964-025-01313-z

[29] Gopaluni RB, Tulsyan A, Chachuat B, Huang B, Lee JM, Amjad F, et al. Modern machine learning tools for monitoring and control of industrial processes: a survey. IFAC-PapersOnLine. 2020;53(2):218–29. doi: 10.1016/j.ifacol.2020.12.126

[30] Lawrence NP, Damarla SK, Kim JW, Tulsyan A, Amjad F, Wang K, et al. Machine learning for industrial sensing and control: A survey and practical perspective. Control Engineering Practice. 2024;145:105841. doi: 10.1016/j.conengprac.2024.105841

[31] Tercan H, Meisen T. Machine learning and deep learning based predictive quality in manufacturing: a systematic review. J Intell Manuf. 2022;33:1879–905. doi: 10.1007/s10845-022-01963-8

[32] Zhang Q, Li S, Wang R. Steel surface defect detection via improved vision transformers. Measurement. 2024;231:114547. doi: 10.1016/j.measurement.2024.114547

[33] Özkan A, Uçar A. Detection of defects on metal surfaces based on deep learning. Appl Sci. 2025;15:1406. doi: 10.3390/app15031406

[34] Kubik C, Hofmann J, Molitor DA, Becker M, Groche P. Reliable machine learning models for manufacturing processes. Int J Adv Manuf Technol. 2025;138:5913–27. doi: 10.1007/s00170-025-15785-4

[35] Yan P, Abdulkadir A, Schatte GA, Aguzzi G, Gha J, Pascher N, et al. Learning actionable world models for industrial process control. arXiv. 2025. doi: 10.48550/arXiv.2503.01411

