# Supplementary material for: Multimodal computer vision and machine learning for predictive monitoring of planar-flow casting of amorphous steel foils
Source: PLoS One. 2026 Nov 3;21(11):e0347230. doi: 10.1371/journal.pone.0347230 (PMC13730325; doi:10.1371/journal.pone.0347230)
Supplement: Supplementary file 1 [file pone.0347230.s001.docx]

# **Supplementary Information**

Multimodal computer vision and machine learning for predictive monitoring of planar-flow casting of amorphous steel foils

**Ritaban Dutta, Michael Kellam and Daniel Liang**

# **Introduction**

This Supplementary Information provides additional quantitative details supporting the experimental design, image-derived descriptors, machine-learning framework, and industrial demonstration presented in the main manuscript. The accompanying tables summarise the experimental operating conditions, descriptor statistics, model configuration, and feature-ablation results. The supplementary figure provides a qualitative industrial demonstration of the applicability of the proposed framework, while the supplementary videos illustrate the automated computer vision workflow for foil tracking, nozzle–wheel gap estimation, and thermal-gradient analysis during planar-flow casting.

The complete processed feature dataset, variable descriptions, and supporting documentation are provided separately as the accompanying **Supplementary_Dataset_PFC_ML** package.

**Table S1. Experimental operating conditions and imaging configuration used for planar-flow casting experiments. The experiments were performed under controlled variations in melt temperature, wheel speed, and nozzle geometry while synchronised RGB and infrared image sequences were acquired for subsequent computer-vision and machine-learning analyses.**

| **Parameter** | **Value / Range** | **Purpose in this study** |
| --- | --- | --- |
| Melt temperature | 1250–1350 °C | Investigate the influence of melt thermal conditions on ribbon formation |
| Wheel speed | 8–12 rpm | Control the cooling rate and solidification behaviour |
| Nozzle geometry | Straight slit / Tapered slit | Evaluate the influence of melt delivery configuration |
| Nozzle slit width | 25–30 mm | Maintain controlled melt flow across experimental conditions |
| Copper wheel diameter | 296 mm | Fixed system parameter for all casting experiments |
| Imaging configuration | Front-view RGB, side-view RGB and infrared (IR) cameras | Simultaneous acquisition of structural and thermal information |
| Synchronised acquisition | Yes | Frame-level alignment between RGB and IR image streams |
| Experimental design | Multiple operating conditions across approximately 200 casting runs | Capture representative process variability for model development |

**Table S2.** Quantitative summary of ridge morphology descriptors extracted from stable and unstable production runs. Statistics are derived from the ridge profiles presented in **Figure 5a–b**. While the average ridge density changed only marginally between operating conditions, unstable production exhibited substantially greater spatial variability and broader ridge-width distributions.

| **Ridge descriptor** | **Stable runs (n = 102)** | **Unstable runs (n = 98)** | **Interpretation** |
| --- | --- | --- | --- |
| Mean ridge width (mm) | 0.26 | 0.36 | Unstable runs produced substantially wider ridges. |
| Median ridge width (mm) | 0.25 | 0.36 | Distribution shifted toward larger ridge widths during unstable operation. |
| Ridge-width IQR (mm) | 0.03 | 0.09 | Greater variability in ridge geometry under unstable conditions. |
| Mean ridge density (ridges mm⁻¹) | 5.05 | 4.98 | Mean density remained similar between operating states. |
| Spatial ridge-density variation | Low | High | Unstable runs exhibited pronounced local fluctuations across foil width. |
| Dominant morphological characteristic | Uniform, closely spaced ridges | Wider, irregular ridge spacing | Indicates deterioration of foil surface uniformity during unstable casting. |

**Table S3.** Summary of thermal descriptors and their relationship with ridge morphology. Quantitative values are derived from the thermal analyses presented in Figure 5c–e. Stable production exhibited more uniform transverse temperature distributions, whereas unstable runs showed significantly larger edge-to-centre thermal gradients that were strongly associated with increased ridge formation.

| **Thermal descriptor** | **Stable runs (n=102)** | **Unstable runs (n=98)** | **Interpretation** |
| --- | --- | --- | --- |
| Peak temperature (°C) | 92.1 ± 2.3 | 89.3 ± 2.7 | Stable runs maintained a slightly higher peak surface temperature. |
| Edge-to-centre temperature ratio | 1.10 (1.03–1.18) | 1.78 (1.60–1.96) | Significantly larger thermal imbalance during unstable production. |
| Thermal imbalance (ΔT, °C) | Lower | Higher | Greater edge cooling produced stronger transverse temperature gradients. |
| Pearson correlation (ridge density vs thermal imbalance) | - | r = 0.78 | Strong positive correlation between ridge density and thermal imbalance. |
| Spearman correlation | - | ρ = 0.76 | Confirms a strong monotonic relationship. |
| Statistical significance | - | p = 2.3 × 10⁻¹⁵ | Relationship highly significant. |

**Table S4.** Principal hyperparameters and training configuration of the feature-based XGBoost and multimodal deep-learning models. The XGBoost model was trained using grouped cross-validation to prevent information leakage between casting runs. The deep-learning fusion framework combined synchronised RGB and infrared image sequences with tabular physics-informed descriptors through a transformer-based fusion module.

| **Model** | **Parameter** | **Value / Setting** |
| --- | --- | --- |
| **XGBoost** | Learning rate | 0.05 |
|  | Maximum tree depth | 6 |
|  | Number of estimators | 500 |
|  | Regularisation (L2, λ) | 1.0 |
|  | Objective function | Regression |
|  | Validation strategy | Group-wise cross-validation by casting run |
| **Deep-learning fusion** | RGB backbone | ResNet-18 |
|  | IR backbone | ResNet-18 |
|  | Feature fusion | Transformer encoder |
|  | Dropout | 0.30 |
|  | Optimiser | Adam |
|  | Initial learning rate | 1 × 10⁻⁴ |
|  | Loss function | Mean squared error (MSE) |
|  | Early stopping | Validation-loss based |
|  | Input modalities | Front-view RGB, side-view RGB, infrared (IR), and tabular descriptors |

# **Figure S1. Qualitative industrial demonstration of the planar-flow casting monitoring framework.** (a) Industrial planar-flow casting system and imaging configuration. (b) Representative side-view of the nozzle–wheel region showing the air gap (g) between the nozzle exit and cooling-wheel surface. (c) Example transverse thermal profile from a representative stable casting sequence. (d) Ridge-width distributions under stable and unstable casting conditions. (e) Relationship between ridge density and the magnitude of the transverse thermal gradient, illustrating the association between ribbon morphology and thermal non-uniformity. (f) Relationship between gap-prediction error and the edge-to-centre temperature (VT) ratio, showing greater prediction variability with increasing thermal non-uniformity. The quantitative values shown in panels (c–f) are derived from the industrial demonstration sequences and are therefore distinct from the laboratory-derived statistics reported in Figure 5 and Tables S2 and S3. Collectively, these examples demonstrate how the proposed framework extracts interpretable geometric, morphological, and thermal characteristics from industrial planar-flow casting imagery. The industrial sequences are presented primarily as a demonstration of framework applicability and consistency of the extracted process descriptors and should not be interpreted as a fully independent external validation of predictive model performance.

**Supplementary Videos**

**Supplementary Video S1**

**Foil tracking and nozzle–wheel gap estimation.**

Representative synchronised side-view video illustrating automated foil tracking, nozzle–wheel gap estimation, and geometric feature extraction during planar-flow casting.

**Supplementary Video S2**

**Thermal-gradient analysis during casting.**

Representative infrared image sequence showing the evolution of temperature distributions across the foil width together with thermal-gradient extraction used for descriptor generation.

The supplementary tables, figure, videos, and accompanying processed dataset collectively provide additional quantitative information and qualitative evidence supporting the methodology and results presented in the main manuscript. Together with the shared feature dataset and variable descriptions, these materials improve transparency and facilitate independent evaluation of the proposed analytical framework.
