# Supplementary material for: Multimodal computer vision and machine learning for predictive monitoring of planar-flow casting of amorphous steel foils
Source: PLoS One. 2026 Nov 3;21(11):e0347230. doi: 10.1371/journal.pone.0347230 (PMC13730325; doi:10.1371/journal.pone.0347230)
Supplement: Supplementary file 2 [file pone.0347230.s004.zip › README.pdf]

README

Supplementary Data for
Multimodal computer vision and machine learning for predictive monitoring of planar-flow casting of amorphous steel foils

Contents

This supplementary package provides the processed feature dataset used for the machine-learning analyses described in the accompanying manuscript.

The package contains:
• **Supplementary_Data.xlsx** – complete processed feature dataset used for model development and evaluation.
• **supplementary_data.csv** – CSV version of the complete processed feature dataset.
• **train.csv** – training subset used in the study.
• **test.csv** – testing subset used in the study.
• **Variable_Description.xlsx** – detailed description of all variables, units and analytical roles.

Dataset Description

The dataset consists of approximately 8,700 aggregated samples derived from synchronised multimodal imaging acquired during planar-flow casting experiments. Each sample contains anonymised sample identifiers, casting-run identifiers, training/testing partition labels, prediction targets, physics-informed image descriptors and process-related variables.

The feature set includes descriptors representing ridge morphology, optical reflection behaviour, thermal characteristics, texture statistics and process variables. Prediction targets include the nozzle–wheel gap and an optical-flow-based flow proxy.

Variable Definitions

Detailed descriptions of every variable, including units and analytical roles, are provided in **Variable_Description.xlsx**.

Analytical Workflow

The feature engineering, preprocessing, model development, validation procedures and statistical analyses are described in detail in the accompanying manuscript. The supplied feature dataset enables independent analysis of the processed features and the development and evaluation of alternative machine-learning models using the same feature set.

Notes

• Sample identifiers are anonymised.
• Casting-run identifiers are retained only to support grouped cross-validation.
• Variable names are consistent with those used throughout the manuscript to facilitate interpretation.
